# Neuronal transcriptome, tau and synapse loss in Alzheimer’s knock-in mice require prion protein

**DOI:** 10.1186/s13195-023-01345-z

**Published:** 2023-11-15

**Authors:** Austin Stoner, Li Fu, LaShae Nicholson, Chao Zheng, Takuya Toyonaga, Joshua Spurrier, Will Laird, Zhengxin Cai, Stephen M. Strittmatter

**Affiliations:** 1 Departments of Neuroscience and Neurology, Yale School of Medicine, New Haven, CT USA; 2 Yale PET Center, Department of Radiology and Biomedical Imaging, Yale School of Medicine, New Haven, CT USA

**Keywords:** Cellular prion protein, Alzheimer knock-in mouse, Transcriptomics, C1q, Synaptic tagging, SV2A PET, snRNA-seq, Tauopathy

## Abstract

**Background:**

Progression of Alzheimer’s disease leads to synapse loss, neural network dysfunction and cognitive failure. Accumulation of protein aggregates and brain immune activation have triggering roles in synaptic failure but the neuronal mechanisms underlying synapse loss are unclear. On the neuronal surface, cellular prion protein (PrP^C^) is known to be a high-affinity binding site for Amyloid-β oligomers (Aβo). However, PrP^C^’s dependence in knock-in AD models for tau accumulation, transcriptomic alterations and imaging biomarkers is unknown.

**Methods:**

The necessity of PrP^C^ was examined as a function of age in homozygous *App*^*NL−G−F*^/*hMapt* double knock-in mice (DKI). Phenotypes of *App*^*NL−G−F*^/*hMapt* mice with a deletion of *Prnp* expression (DKI; *Prnp*^*−/−*^) were compared with DKI mice with intact *Prnp*, mice with a targeted deletion of *Prnp* (*Prnp*^*−/−*^), and mice with intact *Prnp* (WT). Phenotypes examined included behavioral deficits, synapse loss by PET imaging, synapse loss by immunohistology, tau pathology, gliosis, inflammatory markers, and snRNA-seq transcriptomic profiling.

**Results:**

By 9 months age, DKI mice showed learning and memory impairment, but DKI; *Prnp*^*−/−*^ and *Prnp*^*−/−*^ groups were indistinguishable from WT. Synapse loss in DKI brain, measured by [18F]SynVesT-1 SV2A PET or anti-SV2A immunohistology, was prevented by *Prnp* deletion. Accumulation of Tau phosphorylated at aa 217 and 202/205, C1q tagging of synapses, and dystrophic neurites were all increased in DKI mice but each decreased to WT levels with *Prnp* deletion. In contrast, astrogliosis, microgliosis and Aβ levels were unchanged between DKI and DKI; *Prnp*^*−/−*^ groups. Single-nuclei transcriptomics revealed differential expression in neurons and glia of DKI mice relative to WT. For DKI; *Prnp*^*−/−*^ mice, the majority of neuronal genes differentially expressed in DKI mice were no longer significantly altered relative to WT, but most glial DKI-dependent gene expression changes persisted. The DKI-dependent neuronal genes corrected by *Prnp* deletion associated bioinformatically with synaptic function. Additional genes were uniquely altered only in the *Prnp*^*−/−*^ or the DKI; *Prnp*^*−/−*^ groups.

**Conclusions:**

Thus, PrP^C^-dependent synapse loss, phospho-tau accumulation and neuronal gene expression in AD mice can be reversed without clearing Aβ plaque or preventing gliotic reaction. This supports targeting the Aβo-PrP^C^ interaction to prevent Aβo-neurotoxicity and pathologic tau accumulation in AD.

**Supplementary Information:**

The online version contains supplementary material available at 10.1186/s13195-023-01345-z.

## Background

The most common cause of dementia is Alzheimer’s Disease (AD), characterized by the accumulation of amyloid-β (Aβ) plaques and neurofibrillary tangles in the brain [1, 2]. Accumulation of misfolded protein is coupled with synapse loss, inflammation, cognitive decline, and eventually neuronal degeneration and death. Currently, there is no cure for AD, and treatments focus on reducing symptoms and slowing the progression of the disease. Recent clinical trials document some slowing of disease progression during treatment with certain anti-Aβ antibodies [3, 4]. Diffusible oligomeric and protofibrillary species of Aβ (Aβo) are recognized as being most critical to trigger changes in brain function.

Cellular Prion Protein (PrP^C^) was identified in an unbiased expression cloning experiment as a high-affinity oligomer-specific Aβ binding site at the neuronal surface [5–8]. Aβo species interacting with PrP^C^ are detected in mouse models and in human AD brain [9–11]. Numerous studies have examined the requirement for PrP^C^ in mediating effects of Aβo on neurons and mice (reviewed in [8]). Most studies have utilized transgenic mice overexpressing AD-causing variants of APP and PSEN1, and found necessity of PrP^C^ for synapse loss, memory deficits and selective neuronal degeneration using anti-PrP^C^ antibody blockade or PrP antagonists or gene knockout [12–17]. The action of Aβo/PrP^C^ complexes is mediated by aberrant activation of mGluR5 [10, 18–21].

There are limitations and clarifications regarding an Aβo/PrP^C^/mGluR5 model of synaptic damage in AD [8, 22]. First, the in vivo data rely largely on transgenic overexpression of Aβ and not all transgenic models of AD show PrP-dependence [23]. Whether Aβ pathology driven by endogenous expression levels requires this pathway has not been assessed. Second, the transcriptome-wide effect of PrP^C^ on altered gene expression in neurons and glia as a function of AD pathology has not been explored. Third, it is clear that innate immunity and glial reaction have a prominent role in AD [1, 2], but PrP-dependence of interactions between neuronal synaptic mechanisms and glial mechanisms is undefined. Fourth, there is limited or no Tau pathology in the Aβ models, so the connection of PrP^C^ and mGluR5 to tauopathy is unclear, though data from iPSC models as well as studies of Fyn and Pyk2 kinases suggest a link. Finally, the role of PrP^C^ in AD-related synaptic loss has not been monitored using clinically relevant biomarkers.

Here, we utilize a homozygous double knock-in (DKI) mouse model with expression from the endogenous *App* and *Mapt* loci (*App*^*NL−G−F/NL−G−F*^, *Mapt*^*hMAPT/hMAPT*^ [19, 24, 25] to investigate the effects of constitutive *Prnp* loss on AD-related phenotypes. The DKI strain exhibits memory deficits in the presence of PrP^C^, but not with the *Prnp* null allele. Synapse loss measured in DKI mice by PET or by immunohistology is rescued to wild-type levels by *Prnp* knockout. Similarly, tau accumulation, dystrophic neurites and synaptic tagging by C1q were significantly reduced in DKI mice when PrP^C^ was absent. Transcriptional changes associated with the DKI model were found in multiple cell types as a function of age. *Prnp* deletion normalized DKI-dependent dysregulation of most neuronal genes but few glia DKI-dependent dysregulated genes. Gliosis and Aβ plaque in the DKI model were not altered by the absence of *Prnp*. Thus, PrP^C^ is essential for synaptic and neuronal phenotypes in this knock-in AD model, but Aβ accumulation and glial reaction are PrP-independent and dissociable from neuronal changes. The necessity of PrP^C^ for AD-related neuronal phenotypes provides an alternative site for potential intervention distinct from modulation of Aβ accumulation or glial reaction.

## Methods

### Animals

Mice were cared for by the Yale Animal Resource Center and all experiments, including animal husbandry, genotyping, behavioral testing, and euthanasia, were approved by Yale's Institutional Animal Care and Use Committee (IACUC, protocol #07281). Animals were housed in groups of 1–5 mice per cage with access to food and water ad libitum. 12-h light/dark cycles were maintained throughout the duration of animal housing. All strains were maintained on a pure C57Bl6J background after more than 10 backcrosses. The *App*^*NL−G−F/NL−G−F*^, *Mapt*^*hMAPT/hMAPT*^ mice were provided by Drs. Saito and Saido, RIKEN Center for Brain Science [19, 24, 25]. The *Prnp*^*−/−*^ mice, Edinburgh strain, were obtained from Dr. Chesebro of the Rocky Mountain Laboratories [12, 26]. We utilized the following abbreviated strain designations:

$$\begin{array}{l}\mathrm{WT}~\mathrm{for}~{App}^{+/+},\,{Mapt}^{+/+},\,{Prnp}^{+/+}\\\mathrm{DKI}~\mathrm{for}~{App}^{NL-G-F/NL-G-F},\,{Mapt}^{hMAPT/hMAPT},\,{Prnp}^{+/+}\\ {Prnp}^{-/-}\mathrm\;{for}~{App}^{+/+},{Mapt}^{+/+},{Prnp}^{-/-}\\\mathrm{DKI};\,{Prnp}^{-/-}\mathrm\;{for}~{App}^{NL-G-F/NL-G-F},{Mapt}^{hMAPT/hMAPT},{Prnp}^{-/-}\end{array}$$

DKI; *Prnp*^*−/−*^ mice were generated by first crossbreeding DKI mice with *Prnp*^*−/−*^ mice to generate triple-heterozygous *App*^*NL−G−F/*+^*, Mapt*^*hMAPT/*+^*, Prnp*^±^ mice. The triple-heterozygous mice and their offspring were interbred to create a colony of triple-homozygous DKI; *Prnp*^*−/−*^ mice. Mice were genotyped by Transnetyx (Cordova, Tennessee, USA).

### PET imaging of synaptic density

[^18^F]SynVesT-1 was synthesized at the Yale PET Center as utilized for WT and DKI mouse imaging as described [19, 27]. PET measurements were collected on a Focus 220 (Siemens Medical Solutions). PET data were acquired over the period of 30–60 min post intramuscular injection of [^18^F]SynVesT-1, followed by a transmission scan using ^57^Co. Anesthesia was with isoflurane.

The cerebellum was the reference region for *BP*_ND_ values and calculating SUVR_CB_. Differences in SUVR_CB_ between groups were assessed on a voxel-by-voxel basis in Matlab R2018 (Mathworks) with Statistical Parametric Mapping (SPM12). The Z-score for the difference of two groups was calculated in the framework of the general linear model per voxel. Resulting Z-score maps were projected onto the T2-weighted Ma-Benviste-Mirrione mouse brain template.

### Novel object recognition

Mice were tested for novel object recognition as described [13, 20, 21, 28, 29]. Briefly, mice were handled for 5 min a day for 3 days prior to testing to familiarize with the experiment and reduce anxiety. Mice were placed in clean, empty, rectangular, covered rat cages to habituate for 1 h. During acquisition, mice were briefly removed, and 2 identical objects were placed 1 inch from either edge along the long axis of the cage. The objects included: (a wrapped 5 mL plastic syringe or single 15 mL conical tube with an orange cap (3 months), and a large, black binder clip or large glue stick (9 months)). Familiar object choice was pseudorandom. Mice were placed facing perpendicular to the long axis of the cage and a 10-min timer was started. Mice were allowed to explore the two identical objects for the duration of the 10 min, and the time to accrue 30 total seconds of orofacial object exploration was recorded. The objects were removed and either discarded (15 mL conical tube or 5 mL wrapped syringe) or wiped down with 70% ethanol. Mice were left in their cages for 1 h while other mice underwent acquisition.

During testing, one each of the novel and familiar objects were placed on pseudorandom sides of the cage. Orofacial object exploration was timed until 30 total seconds was reached. After testing trials, rat cages were cleaned to eliminate scent cues. The experimenter was blind to novel object identity and genotype. Mice that did not explore both objects or failed to accrue 30 total seconds of orofacial object exploration in less than 6 min during either acquisition or testing were removed from the analysis.

### Morris water maze

Spatial learning and memory were analyzed using the Morris water maze as previously described [20]. Briefly, testing was performed in a circular, open pool ~ 1 m in diameter with the water kept at room temperature (~ 23 °C). Throughout testing and analyses, the experimenter was blinded to genotype. Animals were randomly divided into two cohorts of approximately 40 subjects and swims were conducted in 2 sets of 4 days for 7 consecutive days (day 4 of the first cohort overlapping with day 1 of the second cohort). Acquisition trials consisted of 8 attempts per day for 3 consecutive days, divided into one morning and one afternoon training session of four swims each. For each trial, mice were placed in one of four drop zones facing away from the pool’s center in the quadrant opposite the target quadrant, which contained a submerged plexiglass platform. The order of the drop locations varied across each session. The location of the target quadrant was varied in data acquired at 3 months and 9 months of age. Attempts were considered complete once a mouse located and spent 1 s on the platform. On the first day only, mice were allowed to spend 15 s on the platform until removal from the pool. Additionally, on the first day only, if a mouse did not find the platform within 60 s, it was guided to the platform and allowed to spend 15 s on the platform until removal from the pool.

On day 4, the platform was removed for the probe trial assessment. Mice were placed facing away from the center of the pool between the drop zones furthest from the target quadrant and tracked over 60 s of swim time. Latency to platform during the training swims and percent time spent in the target quadrant during probe trials were recorded on a JVC Everio G-series camcorder and tracked by Panlab’s Smart software. After completion of the probe trial, a visible platform was placed in the target quadrant and trials were administered until latencies stabilized over a maximum number of 15 trials. The latencies for the final 3 trials were averaged.

### Immunohistology of mouse brain sections

Mice were euthanized by CO_2_ inhalation and perfused with ice-cold PBS before decapitation and rapid brain dissection. Right brain hemispheres were drop-fixed in 4% paraformaldehyde for 48 h at 4 °C before transfer to PBS at 4 °C. Right brain hemispheres were sliced into 40 μm cortical brain sections using a Leica WT1000S vibratome and free-floating sections transferred to PBS with 0.05% sodium azide for long-term storage.

For AT8 staining, slices were stained using Alexa Fluor 594 Tyramide SuperBoost kit, Streptavidin (Thermofisher; B40935) according to manufacturer instructions. An antigen retrieval step was performed for pThr217 and PSD-95 single staining prior to the blocking step by incubating slices in 1 × Reveal Decloaker buffer (Biocare Medical; RV 1000 M) for 15 min at 90 °C in an incubator and then 15 min at room temperature. For pThr217 staining, sections were incubated in blocking buffer (1% BSA + 1% Triton X-100 in PBS) for 1 h and then incubated in primary antibodies overnight (~ 24 h) at 4 °C in blocking buffer. For all other stains, sections were blocked in 10% normal horse serum (Jackson ImmunoResearch Laboratories) in PBS + 0.2% Triton X-100 for 1 h and then incubated with primary antibodies overnight (~ 24 h) in 1% normal horse serum in PBS at 4 °C.

The following primary antibodies were used: anti-AT8 (Phospho-TAU (Ser202, Thr205), Biotin; Invitrogen MN1020B; 1:100), anti-LAMP-1 (1D4B) (lysosome-associated membrane protein 1; Santa Cruz sc-19992; 1:500) anti‐IBA1 (ionized calcium‐binding adapter molecule 1; Wako 019‐19,741; 1:250), anti-PSD-95 (for PSD-95 single stain) (postsynaptic density protein 95; Invitrogen 51–6900; 1:250), anti-PSD-95 (for PSD-95/C1Q co-stain) (Millipore MAB1596; 1:200), anti-SV2A (synaptic vesicle glycoprotein 2A; Abcam 32,942; 1:250), anti-NEUN (Fox-3; Millipore ABN91; 1:500), anti-C1Q (Abcam ab182451; 1:1000), anti-pThr217 (phospho-TAU threonine 217; Invitrogen 44–744; 1:200), anti-pS396 (phospho-TAU serine 396; Invitrogen 44-752G; 1:250), anti-PrP (8H4) (prion protein; Abcam ab61409; 1:1000), anti-GFAP (glial fibrillary acidic protein; Abcam ab4674; 1:2000).

Sections were washed in PBS three times for 5 min each and incubated in secondary antibodies in PBS (goat or donkey anti-rabbit, anti-mouse, or anti-rat fluorescent antibodies; Invitrogen Alexa Fluor; 1:500) for 1 h at room temperature in the dark. Sections were washed in PBS three times for 5 min each and then mounted onto glass slides (Superfrost Plus, Fisher Scientific) and cover-slipped with Vectashield (Vector Laboratories H‐1200) antifade aqueous mounting medium.

For Thioflavin S (Sigma; T1892) staining, slices were washed twice for 5 min with 70% ethanol, stained with 0.1% ThioS in 70% ethanol for 15 min at room temperature, and washed twice for 5 min with 70% ethanol before being mounted as previously described. For pThr217 staining, after the three post-secondary PBS washes, slices were briefly dipped in ddH2O and then incubated in CuSO4 buffer (10 mM CuSO 4 in 50 mM ammonium acetate buffer, pH 5) for 15 min at room temperature. Slices were then briefly dipped again in ddH2O, returned to PBS, and mounted as previously described.

### Imaging and analysis of immunohistochemistry

Staining for synaptic proteins with anti-SV2A and anti-PSD-95 antibodies was imaged using a Zeiss LSM 800 confocal microscope with a 63X 1.4 NA oil-immersion lens. The percent area occupied by immunoreactive synaptic puncta from the polymorphic layer of the dentate gyrus was measured as described [12, 19]. For imaging of the tissue stained for AT8, NeuN, GFAP, IBA1, CD68, pS396, pThr217, Lamp1, Aβ, and Thioflavin S, a Zeiss LSM 800 confocal microscope with a 20X 0.8 NA air-objective lens was used. Imaged regions included hippocampus, retrosplenial cortex, and auditory cortex. pS396 was imaged in retrosplenial cortex medially, and auditory cortex laterally. GFAP, Iba1, CD68, Thioflavin S, and Aβ were imaged using a 5 × 3 tile and *z*‐stack through the full slice in the center of the hippocampus with maximum intensity projection. pThr217 was imaged using a 2 × 2 tile and *z*-stack through the full slice in CA1 with maximum intensity projection. AT8 and Lamp1 were imaged using a 2 × 2 tile and *z*-stack through the full slice in medial cortex with maximum intensity projection. The percent area occupied by immunoreactive signal was quantified using ImageJ software. Statistical analysis was based on separate mice, with two slices averaged per mouse [19].

C1q synaptic targeting and PrP/C1q colocalization experiments were completed as described [19]. Briefly, images were acquired using a Zeiss LSM 800 confocal microscope with Airyscan detector and a 63X 1.4 NA oil‐immersion lens. 5-layer z-stacks were acquired from three adjacent locations in CA1 per slice. Images were acquired using a magnification factor of 3 × and optimized XY pixel size and Z pixel dimension per Zeiss Zen software (~ 34 nm and 200 nm, respectively). Following image capture, images were 3D Airyscan processed in Zeiss Zen Blue with an Airyscan filter strength of 6. C1q synaptic targeting and PrP/C1q colocalization were calculated using the JACoP plug-in in ImageJ [30].

Immunofluorescent staining of brainstem tissue utilized tissue from 20-month-old mice. After 24 h of post-fixation, tissue was cut through the midline and sagittally sectioned into 50 μm free-floating sections by vibratome. Sections were collected and stored in serial order. Sections were blocked with PBS containing 0.1% Triton X-100 (American Bio; AB02025) and 1% BSA for 1 h at room temperature. Sections were incubated in rabbit anti-Tyrosine Hydroxylase (Millipore AB152; 1:500) primary antibody diluted in blocking buffer overnight at 4 °C. Slices were washed with PBS and then incubated with goat anti-rabbit Alexa Fluor488 (Thermo Fisher Scientific #A-11008, 1:500) secondary antibody for 1 h at room temperature in the dark. After washing in PBS, sections were dipped into distilled water and then incubated in ammonium acetate with 10 mM CuSO4 (pH 5) for 15 min at room temperature to minimize lipofuscin autofluorescence. Samples were then washed in PBS and incubated with DAPI (1:1000) for 1 h at room temperature. After further washing in PBS, sections were mounted onto glass slides (Superfrost Plus, Fisher Scientific) and coverslipped with Prolong Diamond (ThermoFisher Scientific; P36965) antifade aqueous mounting medium. Tyrosine hydroxylase sections were imaged with a Leica DMi8 confocal microscope with a 10X 0.4 numerical aperture air-objective lens. Seven µm *z*-stacks were acquired from locus coeruleus. Six image slices were *z*-stack projected via maximum orthogonal projection.

For immunohistological analysis of AT8, CD68, D54D2, GFAP, Iba1, Lamp1, ThioS, pS396, bAT8, and pThr217, one field from each of two stained brain sections for each mouse was imaged in the designated areas. Measurements from different sections were averaged to create one value for “n” mice. For synaptic PSD-95 and SV2a staining, and for the C1q/PSD-95 colocalization study, two brain sections were used with two images taken per section in the designated area. For tyrosine hydroxylase staining in the locus coeruleus, five consecutive brain sections containing the nucleus were used with one image per section. All images were acquired, processed, and analyzed by an experimenter blinded to animal genotypes by chip number. Target marker’s fluorescence intensity, and immunoreactive area were quantified using Image J with macros. Positive TH cell numbers were counted blindly to animal genotypes chip number.

### Immunoblotting

Mice at 20-months age were sacrificed via rapid decapitation and hemispheres separated medially on ice using a sharp razor blade. Cortex were microdissected and homogenized on ice with polypropylene pellet pestles in three times the brain tissue weight in RIPA lysis buffer (Millipore 20–188) containing PhosSTOP (Roche; 4,906,845,001) and cOmplete-mini protease inhibitor cocktail (Roche; 11,836,153,001) to extract cytosolic proteins. After centrifugation for 45 min at 100,00 × *g* at 4 °C, the supernatants were collected and boiled in Laemmli sample buffer with 5% β-mercaptoethanol and 1% SDS at 95 °C for 5 min. Sample volumes were normalized to total protein concentration by BCA assay (Thermo Scientific; 23,225).

Supernatants were then subjected to SDS-PAGE through 4–20% Tris–glycine gels (Bio-Rad; 5,671,095) and transferred onto nitrocellulose membranes (Invitrogen #IB23001) using an iBlot 2 Gel Transfer Device (Invitrogen; IB21001). Actin was run on the same gel as the loading control. Nitrocellulose membranes were blocked in blocking buffer for fluorescent western blotting (Rockland; MB-070–010) for 1 h at room temperature and incubated overnight in primary antibodies at 4 °C. Anti-pTau S202/ T205 (AT8) (Thermo Fisher Scientific #MN1020, 1:700), anti-C1Q (Dako A0136; 1:1000), anti-beta Actin (D6A8) Rabbit mAb (Cell Singnaling 8457S; 1:2000) and anti-beta Actin (Abcam ab8226; 1:5000) were used as primaries in this experiment.

Membranes were washed 3 times 5 min each with TBST after primary antibody incubation and then incubated with appropriate secondary antibodies for 1 h at room temperature. Anti-Mouse IRDye 800CW (LI-COR Biosciences #926–32,212, 1:8000) and α-rabbit IRDye 680CW (LI-COR Biosciences; 926–68,023, 1:8000) were used as secondary antibodies. Immunoblots were visualized with a LI-COR Odyssey infrared imaging system. Quantification of band intensities was performed within a linear range of exposure and by ImageJ software. Protein levels were normalized to beta-actin level.

### Single nuclei 10x  genomic sequencing and analysis

Mouse brain tissue collection and mouse brain nuclei isolation for single nuclei RNA-seq were completed as previously described [19]. Briefly, WT, *Prnp*^*−/−*^, DKI, and DKI; *Prnp*^*−/−*^ mice were aged to 40.3 ± 0.7 weeks (10 months) or 143.9 ± 3.5 weeks (20 months) then sacrificed via rapid dissection. Cortical and hippocampal regions from the left-brain hemisphere were microdissected, pooled and immediately frozen on dry ice then stored at -80 °C until nuclei isolation. Sample tissue (50–100 mg wet weight) was homogenized, placed on top of homogenization buffer, and centrifuged for 1 h. Nuclei pellets were obtained, resuspended, and counted on a hemocytometer to inform subsequent dilution or concentration to 700–1200 nuclei/µl for generating single nuclei cDNA libraries.

Barcode incorporated single-nucleus cDNA libraries were constructed using the Chromium Single Cell 3’ Reagents Kit v3 (10 × Genomics) following the manufacturer’s guidelines. Sample libraries, for both 10- and 20-month-aged cohorts were then pooled and underwent batch sequencing on an Illumina NovaSeq 5000 using single indexed paired-end HiSeq sequencing. A sequencing depth of  > 300 million reads was achieved for all samples with an average read depth of 30,000 reads per nuclei. The resulting sequencer BCL files were demultiplexed into FASTQ files. Sequenced samples were then aligned to the mm10-2020-A *Mus musculus* reference genome using the Cell Ranger Count software (pipeline version 6.01, 10 × Genomics), generating barcoded sparse matrices of gene-nuclei raw UMI counts.

Sample gene count matrices were converted and combined into a single anndata object for quality control (QC) and downstream processing with Scanpy (version 1.8.2) [31], a Python-based gene expression analysis toolkit. Genes detected in less than 10 nuclei were discarded. Nuclei with over 5% of UMI counts mapped to mitochondria genes as well as nuclei with less than 50 genes or more than 8000 genes detected were considered outliers and also discarded. In order to perform comparative gene expression analysis, the retained UMI counts were normalized to their library size by scaling the total number of transcripts to 10,000 per nuclei then Log-transformed. In total, across all experimental groups, 206,575 (10 months, *n* = 17 samples) and 219,900 (20 months, *n* = 23 samples) nuclei and 27,479 genes were retained post QC processing and clustering.

Sample snRNA-seq data were clustered using Scanpy’s implementation of *Seurat_v3* [32]. This process first finds nuclei expression state ‘integration anchors’ across sample datasets by identifying common highly variable genes (HVGs) using the *highly_variable_genes* function of Scanpy. In brief, using raw UMI counts, HVGs were identified by ranking the computed normalized variance of each gene across all nuclei. The set of HVGs identified within each sample dataset were then merged to remove batch-specific genes, after which the top 2000 HGVs were annotated within the anndata object. Next, the regression of total library size and mitochondrial transcripts per nuclei was performed with sample-based batch correction using the *Combat* function in Scanpy. The *Scale* function was used to scale the corrected expression matrix to a max unit variance of 10 standard deviations. A neighborhoods graph of the corrected matrix was constructed using the *Neighborhoods* function, with a knn restriction of 10 and the first 25 principal components, computed from HVGs, as input parameters. Integrated Leiden clustering of nuclei into cell-type subgroups was performed from the neighborhoods graph and dimensionally reduced in UMAP space for visualization. To refine the clustering, nuclei doublets were removed from the dataset and integrated Leiden clustering was reiterated. Nuclei were deemed doublets if associated with clusters enriched for cell-specific marker genes of multiple cell types within the same cluster, indicating mixed-cell expression profiles.

Nuclei clusters enriched with a particular set of marker genes were considered to be of the corresponding primary cell type. Using the *rank_genes_groups* function, the top highly differentially expressed marker genes for individual Leiden clusters were tested against the rest using the Wilcoxon Ranked-Sum test. The highest-ranked genes with singular cluster enrichment were deemed marker genes and their cell specificity was varied by literature to determine cluster cell types.

The *rank_genes_groups* function, with Wilcoxon Ranked-Sum test, was also used for differential expressed gene (DEG) analysis between experimental groups for each identified cell type. Only genes expressed in a minimum of 10% of all nuclei post-ranking were considered differentially expressed. Significant DEGs were defined as genes with an adjusted *p-value* (false discovery rate) less than 0.005 and Log-transformed fold change greater than ± 0.25 (Supplemental Table S1).

Gene set enrichment analysis of DEGs from specific cell types was performed using the network visualization software, Cytoscape (version 3.9.1) [33], with application extensions ClueGo (version v2.5.9) [34] and STRING (version 2.0.0) [35]. DEG lists were queried in ClueGo for the overrepresentation of functional and pathway term associations in Gene Ontology (GO) Molecular Functions, Reactome and KEGG ontology databases. A two-sided hypergeometric test with BH-adjusted *p*-value of 0.0005 and 4-gene threshold was used for term enrichment selection. GO fusion of represented terms was used to collapse redundant and related biological themes with more than 50% similarity of associated genes. Filtered term associations were organized as degree-sorted networks for optimal visualization. The same gene sets were separately tested for cellular localization enrichment using GO cellular compartments and protein–protein interaction (PPI) networks using STRING. Highly enriched PPI networks were selected using a confidence score of 0.4 for strength of gene associations with MCL clustering.

### Experimental design and statistical analysis

Each animal was implanted with an implantable electronic ID transponder (Biomedic Data System Inc, NC1043806) encoding a unique chip number. Each experiment was performed and analyzed using the chip Id number with experimenters unaware of genotype.

One-way ANOVA with post hoc Tukey’s multiple comparisons tests, One-way ANOVA with post hoc Dunnett’s multiple comparisons tests, and unpaired two-tailed *t*-tests were performed using GraphPad Prism software, version 9. Group means ± SEM and sample sizes (*n*) are reported in each figure legend. Data were considered statistically significant if *p* < 0.05. For all figures, all statistically significant group differences are labeled. For any given group comparison, the absence of any indication of significant difference implies lack of significance by the applied statistical test.

## Results

###  Aged DKI mouse learning and memory deficits require PrP^C^

**Fig. 1:**
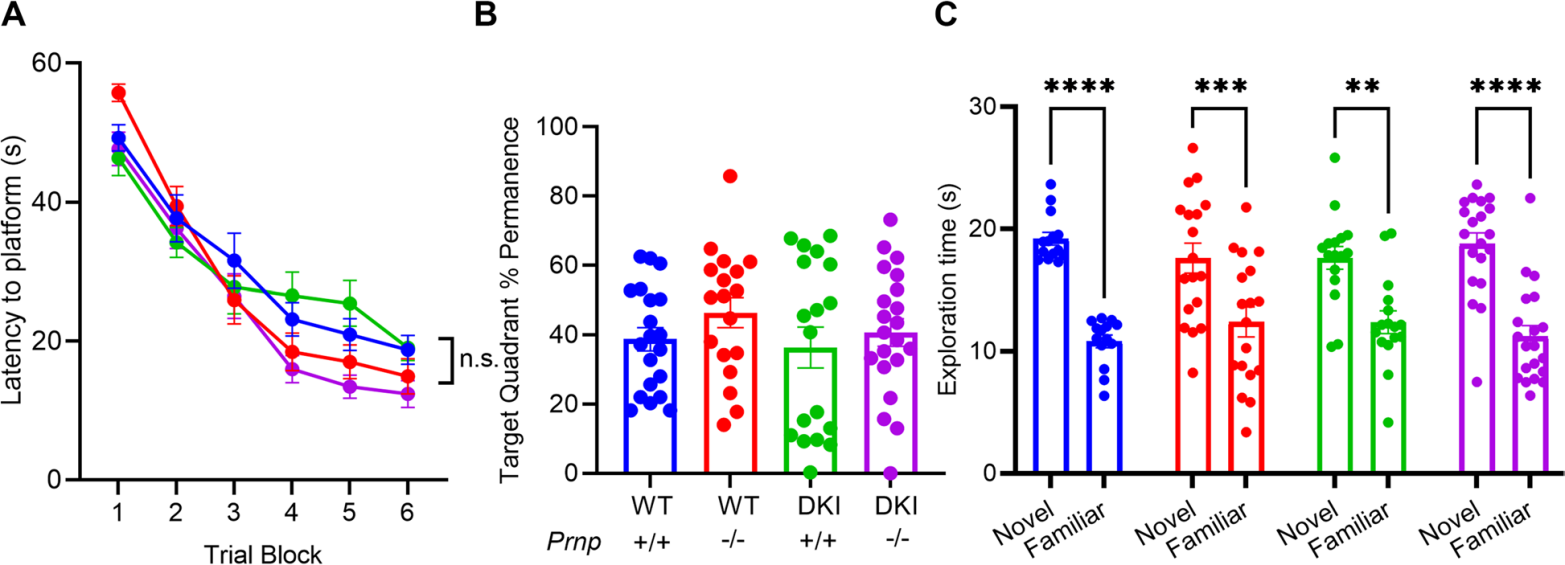
Young Adult DKI mice demonstrate no learning and memory deficit at 3 months. **A** 3‐month‐old WT (blue), *Prnp*^*−/−*^ (red), DKI (green) and DKI; *Prnp*^*−/−*^ (purple) mice completed the MWM to investigate the age of spatial memory deficit. Latency is defined as the average time of 4 trials to find a hidden platform across 6 acquisition sessions. No significant difference was observed in the time to reach the platform during the final acquisition session in any genotype compared to DKI. Data are graphed as mean ± SEM, analyzed by two‐way ANOVA with Dunnett’s multiple comparisons test, *P* > 0.05, *n* = 20 for WT, *n* = 18 for *Prnp*^*−/−*^, *n* = 18 for DKI, and *n* = 21 for DKI; *Prnp*^*−/−*^. **B** 24 h after completing the final acquisition session, all 4 genotypes completed a probe trial. The percent permanence is defined as the fraction of 60 s spent in the quadrant where the platform was during acquisition trials. No significant difference was observed in the percent permanence in any genotype compared to DKI. Data are graphed as mean ± SEM, analyzed by two‐way ANOVA with Dunnett’s multiple comparisons test, *P* > 0.05, *n* = 20 for WT, *n* = 18 for *Prnp*^*−/−*^, *n* = 18 for DKI, and *n* = 21 for DKI; *Prnp*^*−/−*^. **C** 3-month-old WT, *Prnp*^*−/−*^, DKI and DKI; *Prnp*^*−/−*^ mice completed the NOR test. All 4 genotypes preferred to interact with the novel object compared to the familiar object. Data are graphed as mean ± SEM, analyzed by two‐way ANOVA with Sidak's multiple comparisons test, ** *P* < 0.01, ***P < 0.001, ****P < 0.0001, *n* = 14 for WT, *n* = 18 for *Prnp*^*−/−*^, *n* = 16 for DKI, and *n* = 20 for DKI; *Prnp*^*−/−*^

In the DKI model, levels of Aβ plaque are low at 3 months age, are robust at 10 months and are further increased at 20 months (Suppl. Figure 1). The DKI-associated pathology does not alter mouse survival, at least through 12 months (Suppl. Figure 2). We sought to evaluate whether *Prnp* deletion would rescue behavioral deficits in the DKI model. The DKI mice exhibited no significant spatial learning or memory deficits as measured by Morris water maze at 3 months age (Fig. 1A, B), which is consistent with prior studies [19]. In a separate memory test for object recognition, DKI mice showed a similar preference for the novel object as did WT, *Prnp*^*−/−*^, DKI and DKI; *Prnp*^*−/−*^ mice at 3 months age (Fig. 1C). Thus, at this young adult age, DKI mice do not demonstrate significant memory deficits as measured by these two tests.

**Fig. 2:**
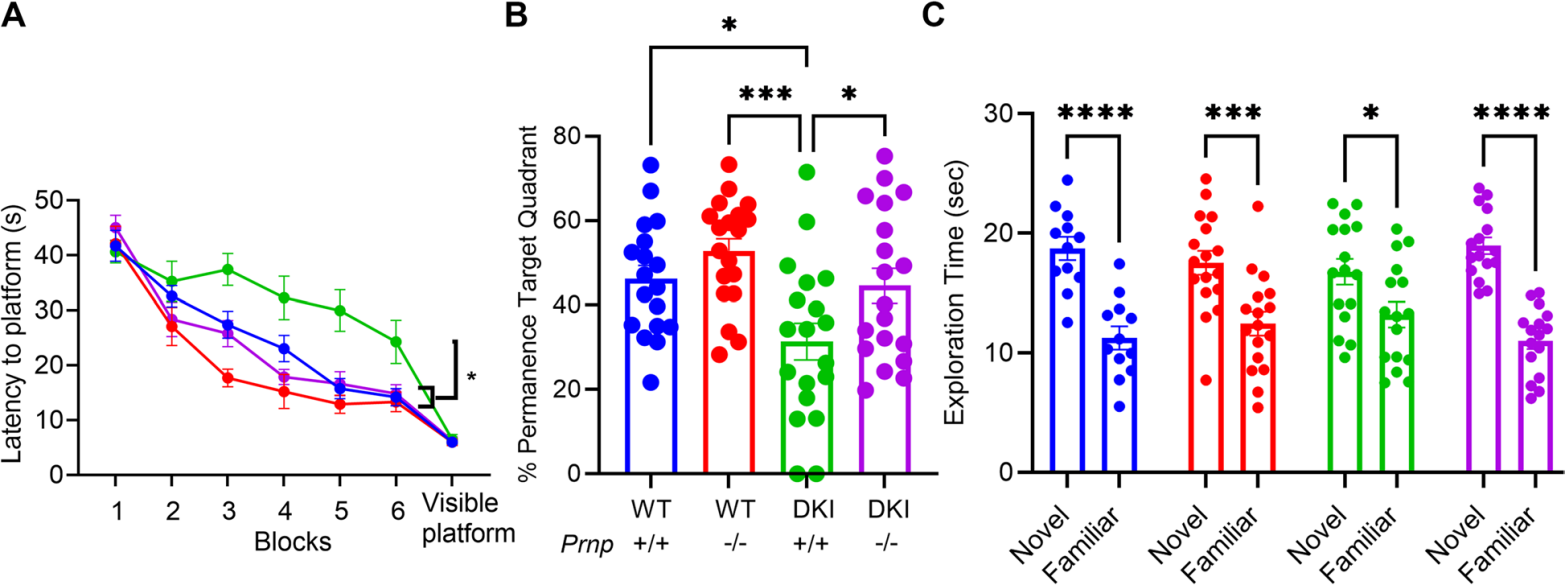
Aged DKI mouse learning and memory impairment at 9 months requires *Prnp* expression. **A** 9‐month‐old WT (blue), *Prnp*^*−/−*^ (red), DKI (green) and DKI; *Prnp*^*−/−*^ (purple) mice repeated the MWM to assess the onset of spatial memory deficit. Latency is defined as the average time of 4 trials to find a hidden platform across 6 acquisition sessions. DKI mice took significantly longer to reach the platform during the final acquisition session compared to WT, *Prnp*^*−/−*^ and DKI; *Prnp*^*−/−*^. The DKI; *Prnp*^*−/−*^ mice showed learning equal to WT levels. To rule out the contribution of visual or motor impairments, latency to a visible platform was measured and no significant difference was observed in any genotype compared to WT. Data are graphed as mean ± SEM, analyzed by two‐way ANOVA with Dunnett’s multiple comparisons test, *P < 0.05, *n* = 18 for WT, *n* = 19 for *Prnp*^*−/−*^, *n* = 19 for DKI, and *n* = 19 for DKI; *Prnp*^*−/−*^. **B** 24 h after completing the final acquisition session, all 4 genotypes completed a probe trial. The percent permanence is defined as the fraction of 60 s spent in the quadrant where the platform was during acquisition trials. DKI mice spent significantly less time in the target quadrant compared to WT, *Prnp*^*−/−*^ and DKI; *Prnp*^*−/−*^ mice. The DKI; *Prnp*^*−/−*^ mice showed rescue equal to WT levels. Data are graphed as mean ± SEM, analyzed by ordinary one-way ANOVA with Dunnett’s multiple comparisons test, *P < 0.05, ***P < 0.001, *n* = 18 for WT, *n* = 19 for *Prnp*^*−/−*^, *n* = 19 for DKI, and *n* = 19 for DKI; *Prnp*^*−/−*^. **C** 9-month-old WT, *Prnp*^*−/−*^, DKI and DKI; *Prnp*^*−/−*^ mice repeated the NOR test. All 4 genotypes preferred to interact with the novel object compared to the familiar object, though DKI; *Prnp*^*−/−*^ mice exhibited a trend towards less novel object recognition. Data are graphed as mean ± SEM, analyzed by two‐way ANOVA with Sidak's multiple comparisons test, * *P* < 0.05, ***P < 0.001, ****P < 0.0001, *n* = 12 for WT, *n* = 17 for *Prnp*^*−/−*^, *n* = 16 for DKI, and *n* = 16 for DKI; *Prnp*^*−/−*^

DKI mice have been shown to demonstrate spatial memory deficits as measured by Morris water maze by 12 months age [19]; however, we sought to test if these mice exhibit observable deficits at an earlier age. We conducted the Morris water maze at 9 months of age on the mice previously tested at 3 months, with the target quadrant placed opposite the 3-month location. DKI mice exhibited learning deficits compared to WT mice, taking significantly longer to find a hidden platform in the final training block (Fig. 2A, *p* < 0.05). These mice also demonstrated an impaired ability to recall the target quadrant location in the probe trial relative to WT mice (Fig. 2B, *p* < 0.05). Both these observed deficits in DKI mice were rescued by *Prnp* gene knockout. All 4 genotypes demonstrated an equivalent latency to locate a visible platform (Fig. 2A), suggesting the differences observed were attributed to spatial memory deficits rather than vision or motor impairment.

We also assessed novel object recognition in these mice at 9 months of age. DKI mice continued to show a significant preference for the novel object that was not statistically different from the other groups (Fig. 2C); however, we observed a trend toward decreased novel object recognition in this group relative to the other groups.

Thus, we observed learning and memory deficits in 9 months old DKI mice during Morris water maze that were fully rescued by *Prnp* deletion. Novel object recognition was not sufficiently impaired in DKI mice at 9 months to assess a requirement for PrP^C^.

### Prnp deletion prevents synapse loss in DKI mice

**Fig. 3:**
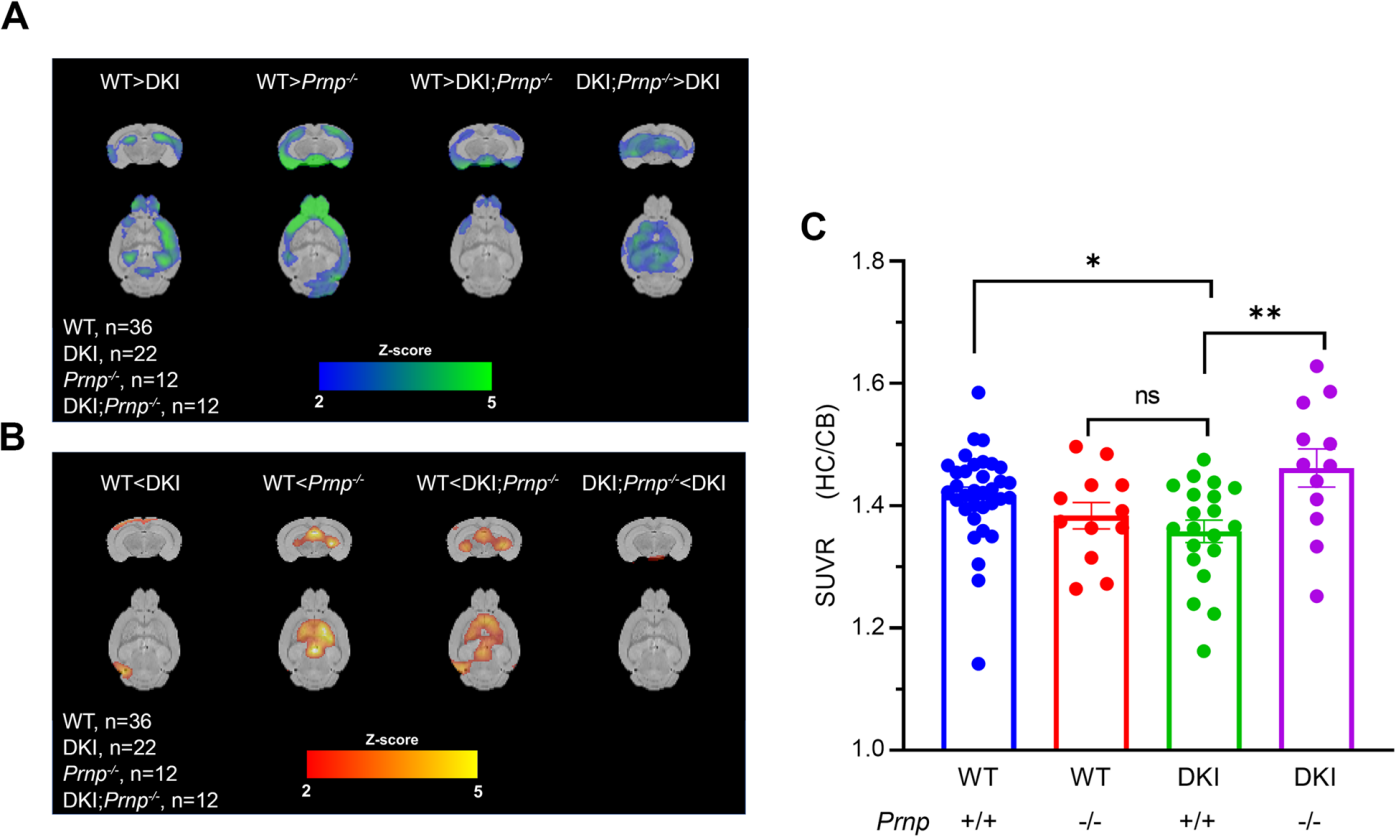
*Prnp* deletion rescues DKI-dependent synapse density reduction measured by SV2A PET. **A**, **B** Voxel-wise analysis t value map comparing averaged [18F] SynVesT-1 PET in 15-month-old mouse brains. Activity is expressed as SUVR (normalized to cerebellum). Color scale represents magnitude of difference in activity in that genotypic comparison. **C** ROI-based comparison of hippocampal [18F]SynVesT-1 SUVR (normalized to cerebellum) in WT, *Prnp*^*−/−*^, DKI and DKI; *Prnp*^*−/−*^ mice. DKI mice demonstrate decreased hippocampal synaptic density compared to WT, while *Prnp* knockout restores hippocampal synapse density in the DKI background. Data are graphed as mean ± SEM, analyzed by ordinary one-way ANOVA with Dunnett’s multiple comparisons test, * *P* < 0.05, **P < 0.01, *n* = 36 for WT, *n* = 12 for *Prnp*^*−/−*^, *n* = 22 for DKI, and *n* = 12 for DKI; *Prnp*^*−/−*^

Synapse loss is an early event in AD progression that correlates strongly with cognitive decline [36, 37]. Thus, we sought to understand if the behavioral deficits observed in DKI mice and subsequent rescue with *Prnp* knockout correspond with changes in synapse density. DKI mice have been shown to exhibit detectable decreases in synapse density at 12 months of age using SV2A PET [19]. At 15 months age, we observed reduced [^18^F]SynVesT-1 binding in the hippocampus of DKI mice relative to WT mice (Fig. 3A,C, *p* < 0.05). Meanwhile, DKI; *Prnp*^*−/−*^ mice demonstrated a full rescue in hippocampal [^18^F]SynVesT-1 standardized uptake value ratio SUVR_CB_ back to WT levels (Fig. 3A,C). We also observed that *Prnp*^*−/−*^ without DKI background showed significant deficits in [^18^F]SynVesT-1 binding in the olfactory bulb region compared to WT mice (Fig. 3A). This data supports previous studies that have implicated PrP^C^ in maintaining mature olfactory sensory neurons [38–40]. Interestingly, despite observing this effect on a wild-type background, DKI; *Prnp*^*−/−*^ mice did not demonstrate synaptic density decrease in olfactory bulb compared to WT mice (Fig. 3A).

**Fig. 4:**
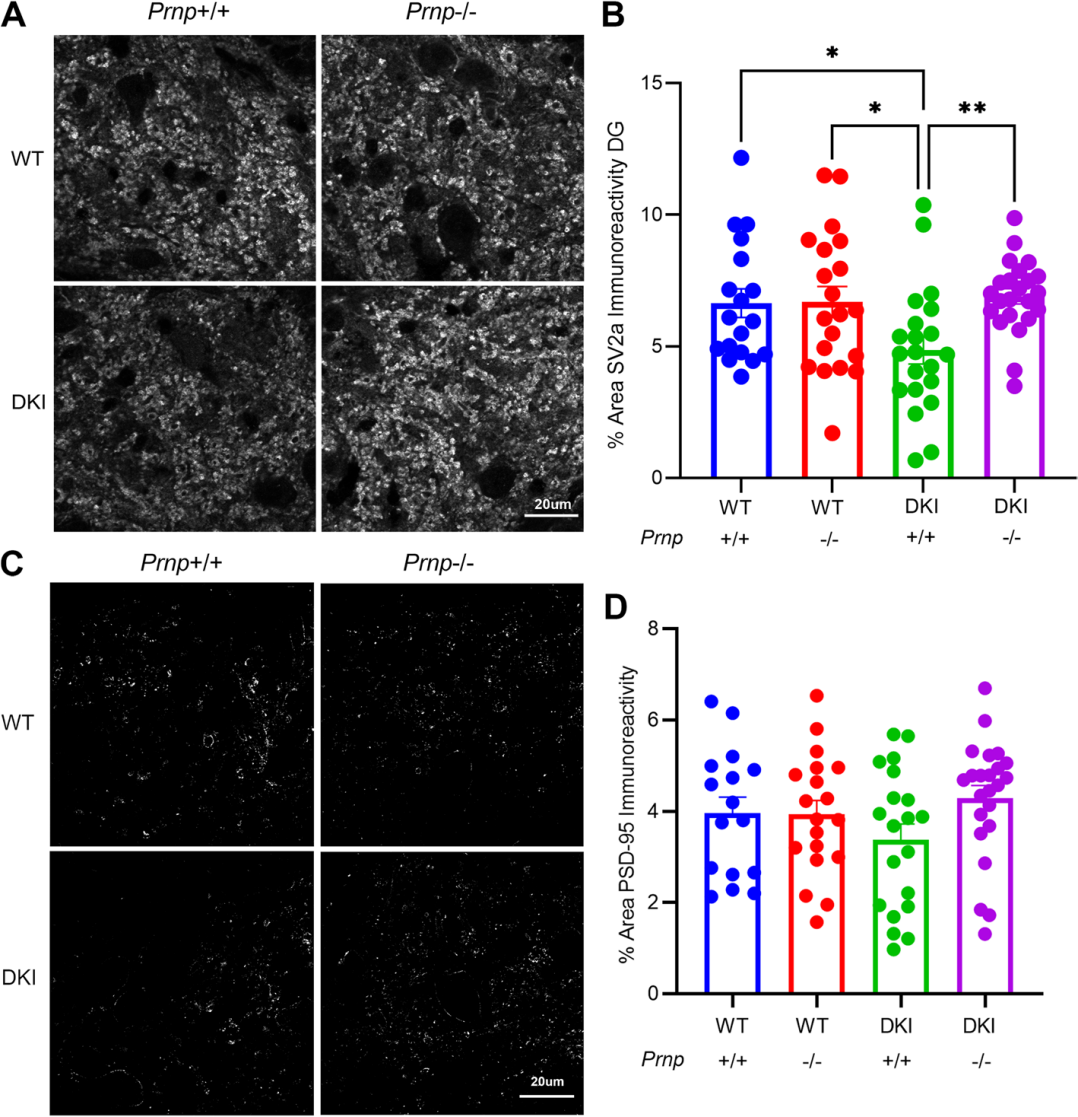
*Prnp* deletion reverses DKI-dependent loss of synaptic immunohistochemical markers. **A** Representative images of staining for the pre-synaptic marker SV2A from the dentate gyrus of 10-month-old WT, *Prnp*^*−/−*^, DKI and DKI; *Prnp*^*−/−*^ mice. Scale bar = 20 μm. **B** Quantification of SV2A immunoreactive area in the dentate gyrus demonstrates a significant decrease in synapse density in DKI mice that is not observed in DKI; *Prnp*^*−/−*^ mice. Data are graphed as mean ± SEM, analyzed by ordinary one-way ANOVA with Dunnett’s multiple comparisons test to DKI, *P < 0.05, **P < 0.01, *n* = 18 for WT, *n* = 20 for *Prnp*^*−/−*^, *n* = 21 for DKI, and *n* = 23 for DKI; *Prnp*^*−/−*^. **C** Representative images of staining for the post-synaptic marker PSD-95 from the dentate gyrus of 10-month-old old WT, *Prnp*^*−/−*^, DKI and DKI; *Prnp*^*−/−*^ mice. Scale bar = 20 μm. **D** Quantification of PSD-95 immunoreactive area in the dentate gyrus demonstrates a trend (*P* = 0.09) towards greater area in DKI; *Prnp*^*−/−*^ mice compared to DKI mice. Data are graphed as mean ± SEM, analyzed by ordinary one-way ANOVA with Dunnett’s multiple comparisons test to DKI, *n* = 16 for WT, *n* = 19 for *Prnp*^*−/−*^, *n* = 20 for DKI, and *n* = 23 for DKI; *Prnp*^*−/−*^

We also tested whether *Prnp* loss prevented synapse loss by analyzing synaptic marker immunostaining. 10-month-old DKI mice exhibited significant (*p* < 0.05) reduction in the presynaptic marker SV2A in dentate gyrus (DG) compared to WT mice, while DKI; *Prnp*^*−/−*^ mice demonstrated a full-rescue to WT levels (Fig. 4A, B). At 10 months age, DKI mice did not exhibit a statistically significant decrease in the postsynaptic marker postsynaptic density protein 95 (PSD-95) in DG compared to WT, though we did observe a trend towards a DG PSD-95 immunoreactivity increase in DKI; *Prnp*^*−/−*^ mice compared to DKI mice (Fig. 4C,D).

Monoaminergic axon and cell degeneration is a pathologic hallmark of AD and is observed in some mouse models [12, 41, 42]. We assessed brain stem neuronal loss in 20-month-old DKI mice using tyrosine hydroxylase (TH) immunoreactivity. We observed a non-significant trend of decreased TH immunoreactivity but not cell number in the locus coeruleus of DKI mice compared to WT (Suppl. Fig. S3). Since there was no robust degeneration phenotype in DKI mice, a role of *Prnp* could not be assessed.

### Phospho-tau accumulation in DKI mice is reduced by Prnp knockout

**Fig. 5:**
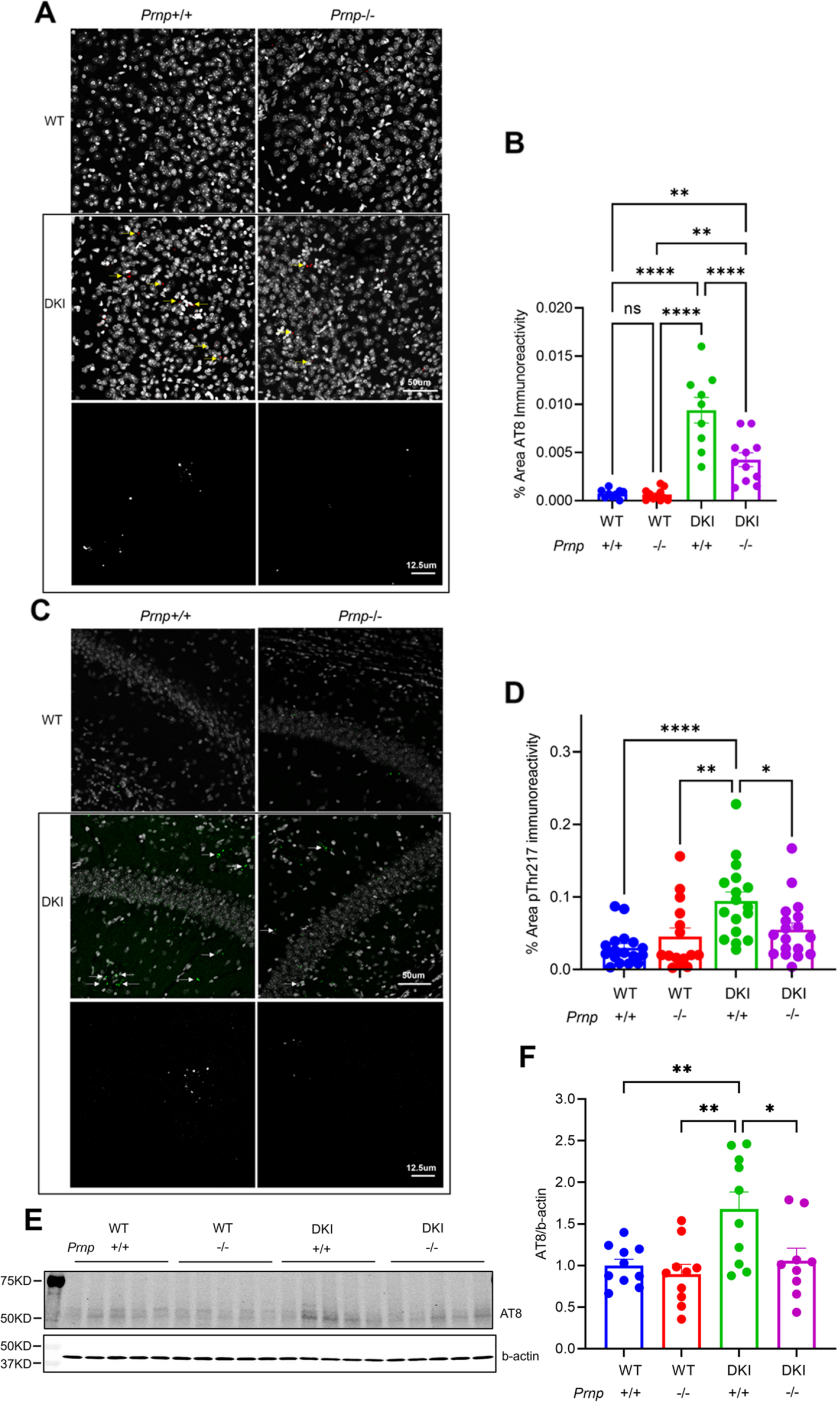
Phospho-tau accumulation in DKI mice is reduced by *Prnp* knockout. **A** Representative images of AT8 (red) staining in the medial cortex of 10-month-old old WT, *Prnp*^*−/−*^, DKI and DKI; *Prnp*^*−/−*^ mice. Gray is DAPI signal, and AT8 inclusions are indicated by yellow arrows in the upper four panels with scale bar = 50 μm. The lower two panels show the AT8 signal only in gray scale at higher magnification with scale bar = 12.5 µm. **B** Quantification of AT8 immunoreactive area in retrosplenial cortex demonstrates a significant increase in phospho-tau accumulation in DKI mice compared to WT. There is a significant decrease in DKI; *Prnp*^*−/−*^ mice relative to DKI. Data are graphed as mean ± SEM, analyzed by ordinary one-way ANOVA with Tukey’s multiple comparisons test, **P < 0.01,****P < 0.0001, *n* = 9 for WT, *n* = 11 for *Prnp*^*−/−*^, *n* = 9 for DKI, and *n* = 11 for DKI; *Prnp*^*−/−*^. **C** Representative images of pThr217 (green) staining in the CA1 of hippocampus of 10-month-old WT, *Prnp*^*−/−*^, DKI and DKI; *Prnp*^*−/−*^ mice. Gray is DAPI signal, and pThr217 inclusions are indicated by arrows in the upper four panels with scale bar = 50 μm. The lower two panels show the pThr217 signal only in gray scale at higher magnification with scale bar = 12.5 µm. **D** Quantification of pThr217 immunoreactive area in CA1 demonstrates a significant increase in phospho-tau accumulation in DKI mice compared to WT. The DKI; *Prnp*^*−/−*^ samples show a significant decrease relative to DKI. Data are graphed as mean ± SEM, analyzed by ordinary one-way ANOVA with Dunnett’s multiple comparisons test, *P < 0.05, **P < 0.01,****P < 0.0001, *n* = 17 for WT, *n* = 15 for *Prnp*^*−/−*^, *n* = 17 for DKI, and *n* = 18 for DKI; *Prnp*^*−/−*^. **E** Immunoblot image of cortical brain lysates stained for AT8 from 20-month-old WT, *Prnp*^*−/−*^, DKI and DKI; *Prnp*^*−/−*^ mice. A cropped region of the blot with migration of appropriate Mol Wt markers is shown at left. **F** Quantification of AT8 antibody immunoreactive protein levels by densitometric analysis. The level of Tau phosphorylated at S202/T205 (AT8) is significantly greater in DKI mice compared to WT or *Prnp*^*−/−*^. The DKI; *Prnp*^*−/−*^ AT8 level is less than that from DKI. Data are graphed as mean ± SEM, analyzed by ordinary one-way ANOVA with Dunnett’s multiple comparisons test, *P < 0.05, **P < 0.01, *n* = 10 for WT, *n* = 10 for *Prnp*^*−/−*^, *n* = 10 for DKI, and *n* = 9 for DKI; *Prnp*^*−/−*^

The accumulation of hyperphosphorylated Tau and neurofibrillary tangles are pathological hallmarks of AD triggered by Aβ pathology [1, 2, 43]. The DKI model replaces murine *Mapt* with human *MAPT* and demonstrates increased levels in certain Tau epitopes, including AT8, pThr217, and pS396 [19, 25]. We assessed the influence of *Prnp* expression on phosphorylated Tau levels in DKI mice by immunohistochemistry and biochemistry. In 10-month-old animals, we detected a significant increase in AT8 immunoreactivity in retrosplenial cortex (*p* < 0.0001) and pThr217 immunoreactivity in the CA1 region of hippocampus (*p* < 0.0001) for DKI compared to WT (Fig. 5A-D). The DKI; *Prnp*^*−/−*^ tissue showed a significant decrease of both AT8 and pThr217 immunoreactivity relative to DKI, with pThr217 immunoreactivity indistinguishable from WT levels (Fig. 5A-D). Biochemical analysis of 20-month-old mice cortical tissue revealed significantly elevated AT8 level in DKI mice relative to WT, with a significant decrease of DKI; *Prnp*^*−/−*^ levels relative to DKI to a level equaling WT values (Fig. 5E-F). Thus, the accumulation of these AD-associated phospho-Tau epitopes depends on PrP^C^ in DKI mice.

The DKI mice also exhibited increased pS396 immunoreactivity in cerebral cortex (*p* < 0.0001) compared to WT (Suppl. Fig. S4A-D) [19]. However, the enhanced pS396 signal in DKI mice co-localizes exclusively with a marker for oligodendrocytes (Olig2) rather than a neuronal marker (NeuN) (Suppl. Fig. S4E, F). The effect of *Prnp* deletion was complicated in these non-neuronal cells. In cerebral cortex, there was a slight increase for *Prnp*^*−/−*^ mice in oligodendrocyte-lineage cells. For the DKI; *Prnp*^*−/−*^ samples, the levels were similar to DKI (Suppl. Fig. S4A-D).

### Prnp loss reduces dystrophic neurites around neuritic plaques without altering Aβ accumulation

**Fig. 6:**
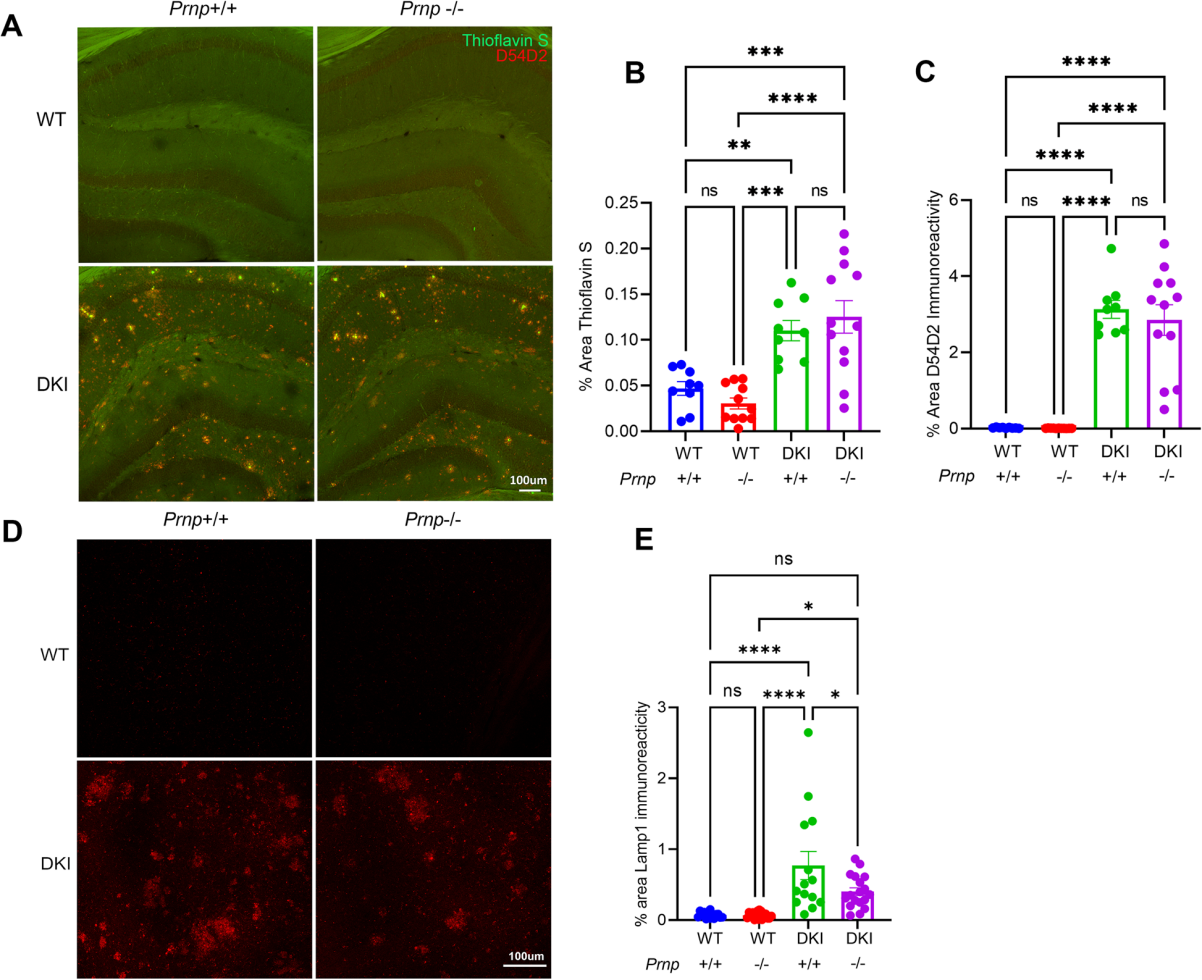
*Prnp* deletion does not alter Aβ accumulation in DKI mice but reduces periplaque dystrophic neurites. **A** Representative images of Thioflavin S (green) and D54D2 anti- Aβ antibody (red) staining from the hippocampus of 10-month-old WT, *Prnp*^*−/−*^, DKI and DKI; *Prnp*^*−/−*^ mice. Scale bar = 100 μm. **B** Quantification of Thioflavin S stained area in hippocampus demonstrates a significant increase in dense core plaque load in DKI mice compared to WT, but no difference between DKI and DKI; *Prnp*^*−/−*^. Data are graphed as mean ± SEM analyzed by ordinary one-way ANOVA with Tukey’s multiple comparisons test, **P < 0.01, ***P < 0.001, ****P < 0.0001, *n* = 9 for WT, *n* = 11 for *Prnp*^*−/−*^, *n* = 9 for DKI, *n* = 12 for DKI; *Prnp*^*−/−*^. **C** Quantification of D52D2 immunoreactive area in hippocampus documents a significant increase in Aβ levels in DKI mice compared to WT, but no difference between DKI and DKI; *Prnp*^*−/−*^. Data are graphed as mean ± SEM, analyzed by ordinary one-way ANOVA with Tukey’s multiple comparisons test, ****P < 0.0001, *n* = 9 for WT, *n* = 11 for *Prnp*^*−/−*^, *n* = 9 for DKI, and *n* = 12 for DKI; *Prnp*^*−/−*^. **D** Representative images of anti-Lamp1 (red) immunostaining in medial cortex of 10-month-old WT, *Prnp*^*−/−*^, DKI and DKI; *Prnp*^*−/−*^ mice. Scale bar = 100 μm. **E** Quantification of the area of Lamp1 immunoreactive accumulation in medial cortex demonstrates a significant increase of dystrophic neurites in DKI mice relative to WT. The accumulation of dystrophic neurites is less pronounced in DKI; *Prnp*^*−/−*^ mice than in DKI mice. Data are graphed as mean ± SEM, analyzed by ordinary one-way ANOVA with Tukey’s multiple comparisons test, *P < 0.05, ****P < 0.0001, *n* = 14 for WT, *n* = 17 for *Prnp*^*−/−*^, *n* = 14 for DKI, and *n* = 18 for DKI; *Prnp*^*−/−*^

The overarching hypothesis to be tested is that PrP^C^ is required for deleterious neuronal phenotypes as a receptor, but not for the production or accumulation of Aβ itself. Therefore, we measured dense core Aβ plaque load and Aβ accumulation with Thioflavin S and D54D2 anti-Aβ antibody staining, respectively (Fig. 6A-C, Suppl. Figure 5). Parenthetically, the area of dense Aβ plaque detected by thioflavin was substantially less than the area detected with anti-Aβ antibody staining in the DKI model, regardless of *Prnp* genotype. *Prnp* knockout had no observable effect on Aβ accumulation in DKI mice (Fig. 6A-C, Suppl. Figure 5). Thus, any PrP^C^-mediated neuronal phenotypes in DKI mice occur independently of Aβ accumulation, consistent with the hypothesis of PrP^C^ as a synaptic binding site for Aβ oligomers.

The presence of Aβ plaques in the AD brain is associated with local activation and increased density of microglia as well as astrocytosis. In the aged DKI brain, evidence for astrocytosis, microgliosis and microglial activation is clear (Suppl. Fig. S6). Paralleling the constant level of Aβ accumulation with and without PrP^C^, the deletion of *Prnp* had no detectable effect on markers of gliosis in DKI mice.

Dystrophic neurites in the vicinity of Aβ plaques are rich in lysosomal and autophagic markers and are thought to contribute to AD pathogenesis [44–46]. Therefore, we assessed whether the periplaque accumulation of neuronal organelles, which occurs in vicinity of high Aβo concentration, might require PrP^C^. We visualized dystrophic neurites by staining 10-month-old AD mouse brains with antibodies directed against lysosome-associated membrane protein-1 (LAMP-1). The DKI brain showed increased LAMP-1 immunoreactive clusters in medial cortex compared to WT mice (*p* < 0.0001) consistent with a periplaque pattern, and *Prnp* knockout significantly decreased LAMP-1 accumulation on the DKI background (*p* < 0.05) (Fig. 6D, E, Suppl. Fig. S7). Thus, while *Prnp* loss does not impact plaque burden or Aβ staining, PrP^C^ contributes to the formation of dystrophic neurites and presumably neuronal lysosomal dysfunction.

### PrP^C^ is required for the localization of C1q to PSD-95 puncta

**Fig. 7:**
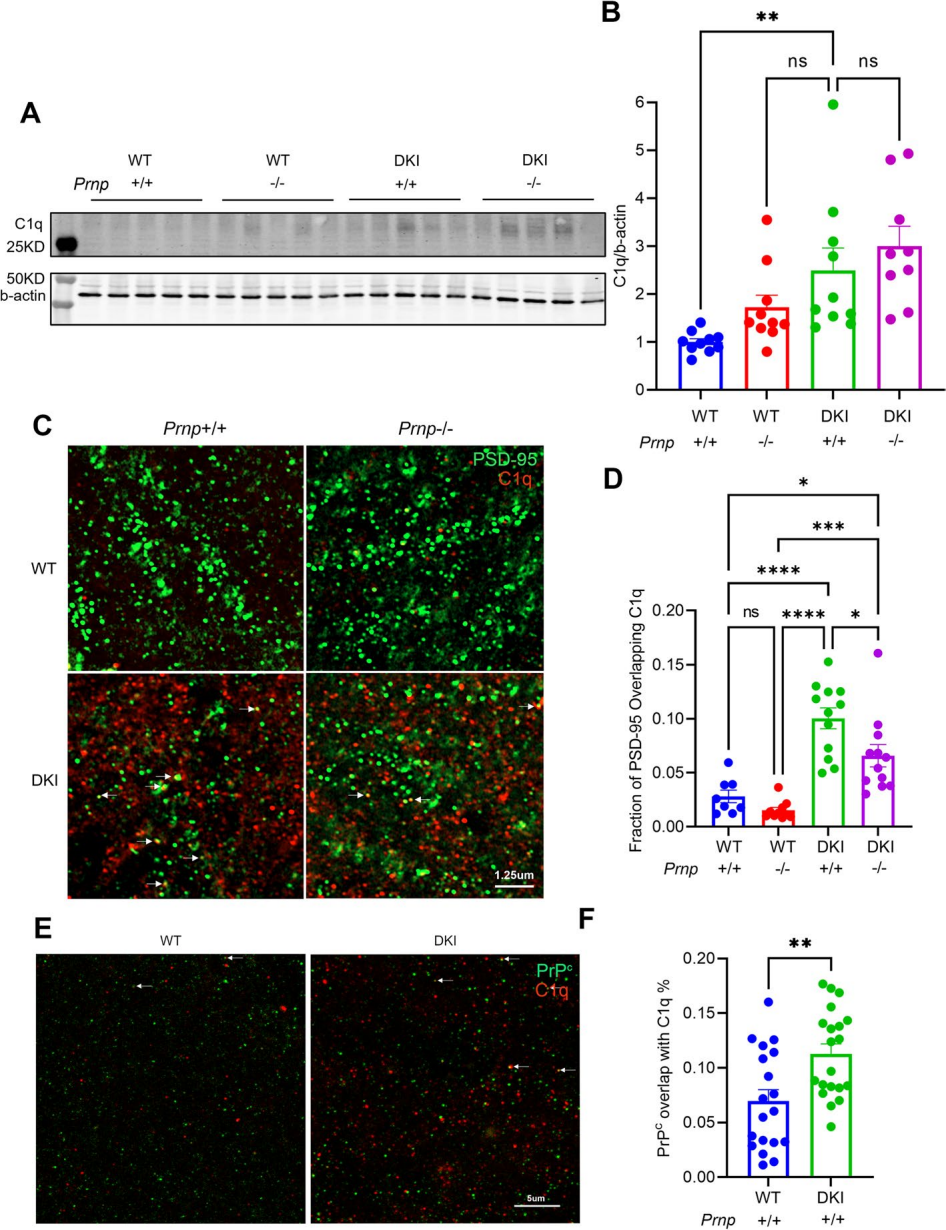
PrP^C^ mediates the localization of C1q to PSD-95 puncta without altering C1q levels. **A** Anti-C1q immunoblot image of cortical brain lysates from 20-month-old WT, *Prnp*^*−/−*^, DKI and DKI; *Prnp*^*−/−*^ mice. A cropped region of the blot with migration of appropriate Mol Wt markers is shown at left. **B** Quantification of C1q protein levels from densitometric analysis of immunoblots as in A. DKI animals exhibit significantly increased C1q expression compared to WT. The C1q level in DKI; *Prnp*^*−/−*^ mice does not differ from DKI group values. Data are graphed as mean ± SEM, analyzed by ordinary one-way ANOVA with Dunnett’s multiple comparisons test, *P* > .05, **P < .01, *n* = 10 for WT, *n* = 10 for *Prnp*^*−/−*^, *n* = 10 for DKI, and *n* = 9 for DKI; *Prnp*^*−/−*^. **C** Representative images of PSD-95 (green) and C1q (red) immunoreactivity in sections of CA1 hippocampus of 10-month-old old WT, *Prnp*^*−/−*^, DKI and DKI; *Prnp*^*−/−*^ mice. Scale bar = 1.25 μm. **D** Quantification of the fraction of PSD-95 immunoreactivity overlapping C1q demonstrates a significant increase in the marking of synapses by C1q in DKI animals compared to WT and WT ^prnp−/−^ mice that are significantly decreased by Prnp gene knockout. Data are graphed as mean ± SEM, analyzed by ordinary one-way ANOVA with Tukey’s multiple comparisons test, *P < 0.05, ***P < 0.001, ****P < 0.0001, *n* = 8 for WT, *n* = 10 for *Prnp*^*−/−*^, *n* = 12 for DKI, and *n* = 12 for DKI; *Prnp*^*−/−*^. **E** Representative images of PrP^C^ (green) and C1q (red) immunoreactivity in CA1 of 10-month-old WT and DKI mice. Scale bar = 5 μm. **F** Quantification of the fraction of PrP^C^ immunoreactivity overlapping C1q demonstrates a significant increase in the colocalization of PrP^C^ and C1q in DKI samples compared to WT brain. Data are graphed as mean ± SEM, analyzed by unpaired t test, **P < 0.01, *n* = 19 for WT, *n* = 20 for DKI

Microglia-mediated phagocytosis of synapses is mediated by C1q and is implicated in AD synapse loss with complement tagging synapses for removal [19, 47, 48]. We previously showed that DKI mice have increased localization of complement C1q protein to synapses compared with WT [19]. Here, we assessed the necessity of PrP^C^ for this synaptic tagging phenomenon in 10-month-old and 20-month-old mice. The DKI brain contains greater immunoblot signal for C1q than WT, but *Prnp* deletion has no impact on overall C1q levels in DKI or WT mice (Fig. 7A, B). This is consistent with the unchanged levels of gliosis and Aβ accumulation. Next, we considered whether PrP^C^ alters the efficiency of C1q tagging of synapses by colocalization with PSD-95. The fraction of PSD-95 immunostaining overlapping C1q immunostaining was significantly increased in DKI mice compared to WT, while *Prnp* knockout resulted in significantly reduced PSD-95/C1q overlap on the DKI background (*p* < 0.05) (Fig. 7C, D).

The reduction in synaptic tagging may reflect C1q interaction with synaptic complexes containing PrP^C^. C1q has been shown to complex with cytotoxic prion protein oligomers and to mediate the development of prion disease [49, 50]. We assessed the colocalization of PrP^C^ with C1q in 10-month-old mouse brain tissue. Immunostaining demonstrated an increase in the fraction of PrP^C^ overlapping C1q in DKI mice compared to WT (Fig. 7E, F). This data supports a potential role for PrP^C^ in the tagging of synapses by C1q, and is likely to contribute to the mechanism by which *Prnp* deletion rescues synapse loss in the DKI model.

### Transcriptomic analysis of cellular changes in DKI mice with and without PrP^C^

**Fig. 8:**
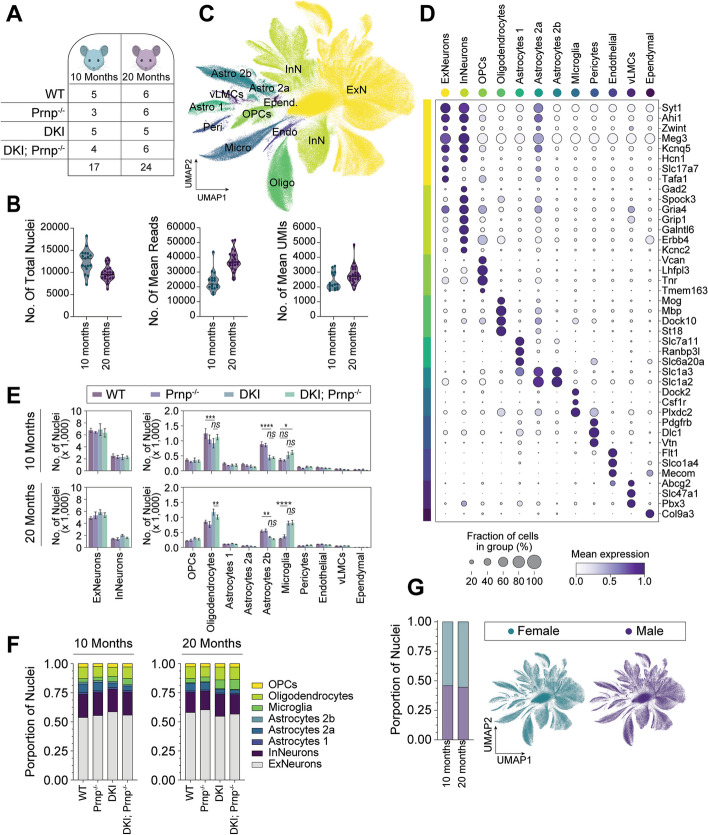
Integration and cell-type classification of age-grouped snRNA-seq data sets. **A** Schematic of treatment group and cohort sample sizes by age group. **B**) Number of total nuclei per sample (left), mean reads per nuclei per sample (middle), and mean unique genes detected per nuclei per sample (right) from single-nucleus RNA-seq (snRNA-seq) of 10-month and 20-month cohorts. **D** Dot plot showing the percentage of nuclei and the scaled mean expression of cell-type classification markers used to identify the UMAP clusters in (**C**). **E** Comparative mean nuclei frequency of cluster cell types representing individual samples of WT, *Prnp*^*−/−*^, DKI, or DKI; *Prnp*^*−/−*^ genotypes. Data presented as mean + SEM from individual samples by their respective genotype and compared by mixed model test with Tukey correction. ns = non-significant, *P < 0.0332, **P < 0.0021, *** < 0.0002,****P < 0.0001. **F** Nuclei proportions for neuronal and glial cell types across groups for 10-month and 20-month cohorts. **G** Nuclei proportions and UMAP projection of male and female representation within the age-group integrated snRNA-seq-data

To provide greater insight into the cellular and molecular mechanism whereby *Prnp* deletion rescues multiple aspects of neuronal function in the DKI model, we conducted deep single-nuclei RNA sequencing (snRNA-seq) and transcriptomic analysis of WT and DKI mice with and without *Prnp* deletion at 10 and 20 months of age (Fig. 8). Cortical plus hippocampal tissue from male and female mouse brain was processed to yield 10,000 nuclei per sample, and the mean sequencing depth was 30,000 reads per nuclei (Fig. 8A, B). The integrated and batch-corrected sequences were clustered in UMAP space (Fig. 8C). Cell clusters were identified by the expression of top cell-type specific classification markers (Fig. 8D). In addition to several excitatory (ExNeurons, ExN) and inhibitory neurons (InNeurons, InN) clusters, three distinct clusters of astrocyte (Astro) cell types were also identified (Fig. 8C, D).

Nuclei of all cell types were present in both age groups and sexes (Fig. 8E) with male and female nuclei proportions equally represented across all cluster cell types (Fig. 8G). Neuronal nuclei proportions did not vary across groups (Fig. 8E-F), reflecting an absence of neuronal loss in this model. In contrast, genotype differences in glial cell proportions were observed (Fig. 8E-F). At both 10 and 20 months, there was a ~ 50% decrease in the sub-group proportion of astrocyte 2b in DKI groups as compared with WT, with and without *Prnp*. The overall proportion of astrocyte cell types was also decreased for all genotypes at 20 months as compared to 10 months. For microglia, cell portions were higher in DKI groups as compared to WT at 10 months, with a 50% increase in microglia cell counts at 20 months. Oligodendrocyte nuclei counts were also higher in DKI groups at 20 months as compared to WT. The glial cell proportion differences occurred in DKI groups with and without *Prnp* deletion, indicating that these AD and age-dependent changes in glial number are independent of PrP^C^. This is consistent with the histological analyses described above.

**Fig. 9:**
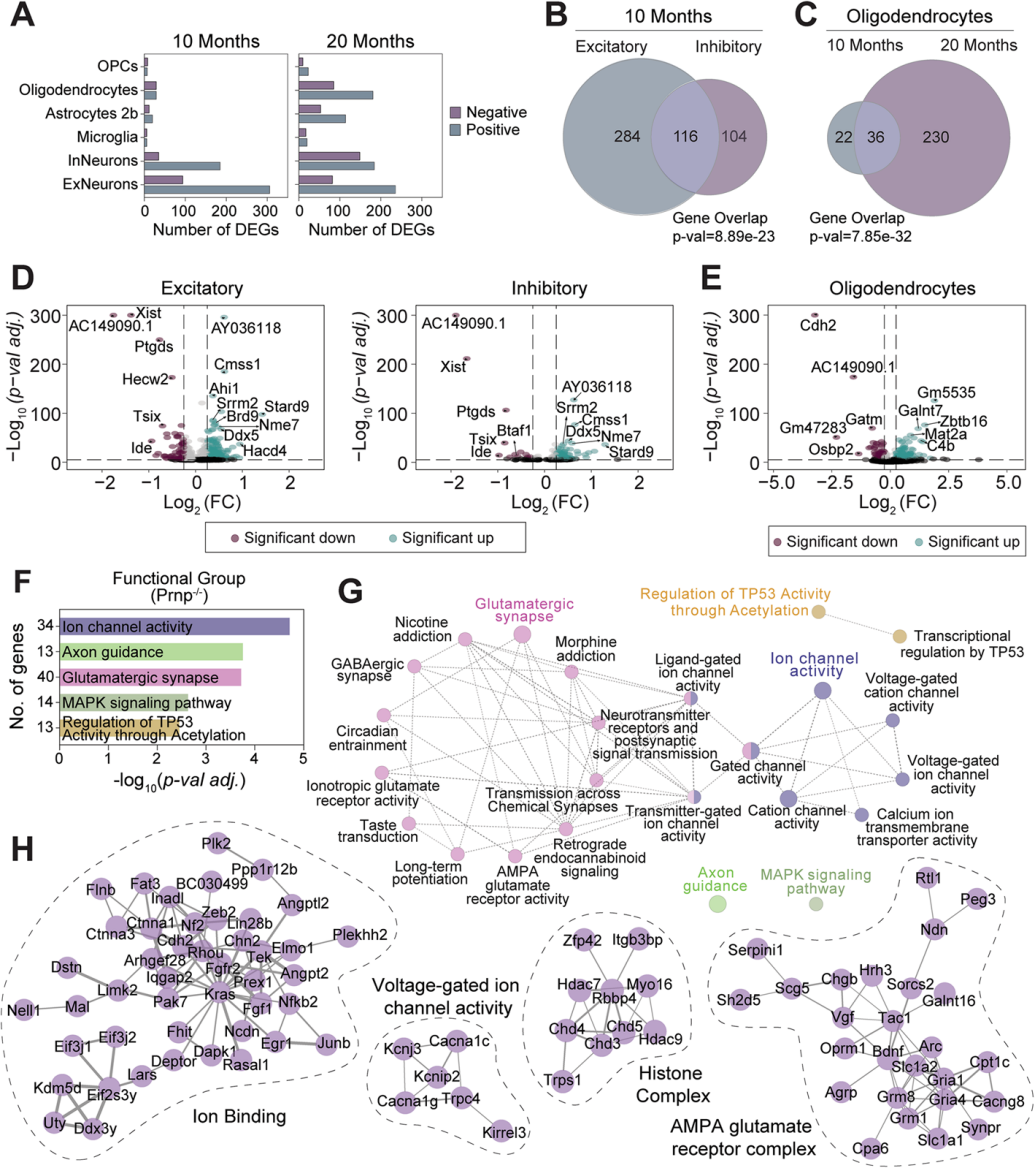
PrP^C^-dependent gene expression profile in neuronal and glial cell populations. **A** The number of positively and negatively differently expressed genes (DEGs) in neuronal and glial cell sub-populations of 10-months (left) and 20-months (right) in *Prnp*^*−/−*^ mice as compared to WT. **B** Venn diagram demonstrating the number of shared DEGs between excitatory and inhibitory neuronal cell populations of 10-month-old *Prnp*^*−/−*^ mice. **C** Venn diagram of significant DEGs in oligodendrocytes shared between 10- and 20-months old *Prnp*^*−/−*^ mice. The statistical significance of gene overlap shown in B,C was assessed by Fisher’s exact test relative to the number of DEGs. **D** Volcano plots representing the gene expression changes in excitatory (left) and inhibitory (right) cells population showing the statistical significance (Log_10_*(p-val adj.),* y-axis) vs the Log_2_ Fold Change (Log_2_(FC), x-axis) of the *Prnp*^*−/−*^ group as compared to WT; vertical dashed lines indicate ± 0.25 Log_2_(FC). A selection of significant PrP^C^-associated genes is labeled. DEGs are deemed significant if they exhibit an absolute Log_2_(FC) > 0.25, with *p* < *0.005* (Wilcoxon rank sum test) and are colored purple (downregulated) or teal (upregulated). For the full gene list, refer to Suppl. Table S1. **E** Volcano plot of gene expression changes identified in 20-month oligodendrocyte cell populations. **F**, **G** Pathway enrichment analysis of pooled significant excitatory and inhibitory DEGs between *Prnp*^*−/−*^ versus WT mice at 10 months. **G** Enrichment terms (nodes), resulting from GO Molecular Functions, KEGG, and REAC Pathway analysis, organized into functional network groups which are linked by shared gene associations (edges). The intra-group significant terms (leading terms) are color highlighted with corresponding *p*-value and total functional group gene association count plotted in (**F**). **H** Protein–Protein interaction (PPI) evidence of gene set enrichment analysis shown in (**F**, **G**). Genes with stronger associations are connected by thicker lines with gene groups encircled based on shared functional pathways

Our analysis focused on transcriptomic changes within cell types, focusing first on the role of *Prnp* under non-pathological conditions (Fig. 9). *Prnp* deletion generated several differentially expressed genes (DEGs) in ExNeurons and InNeurons as compared to WT at both 10 and 20 months (Fig. 9A, B). Volcano plots revealed genes similarly up or downregulated in both ExNeurons and InNeurons (Fig. 9D), indicating a common functional role of *Prnp* in all neuronal types. While the number of neuronal DEGs remained largely unchanged between age groups, there was an age-dependent increase in *Prnp*-associated DEGs in glial (Fig. 9A). Interestingly, the genetic dysregulation induced by *Prnp* deletion was greatest in oligodendrocytes, increasing tenfold from 10 and 20 months, as compared to microglia and astrocytes (Fig. 9A,C). Of the *Prnp*-associated DEGs in WT oligodendrocytes, an adhesion molecule for neuronal cell contact, Cdh2, was most significantly downregulated (Fig. 9E, see Suppl. Table S1 for full list).

The *Prnp* deletion DEG list of 10-Month ExNeurons and InNeurons was analyzed for cellular and functional enrichment with ClueGo [34]. Gene Ontology (GO) cellular compartment analysis detected highly enriched terms related to neuronal synapses, specifically post-synaptic membrane structures (Suppl. Fig. S8, Suppl. Table S2). Gene set enrichment of functional network associations identified pathways related to glutamatergic synapses and ion channel activity (Fig. 9F-G, Suppl. Table S2), both contained strongly connected protein–protein interaction (PPI) networks involved in synaptic signal transduction (Fig. 9E). Thus, in the absence of AD-related changes, certain aspects of synaptic function are modulated by constitutive PrP^C^ loss in adult mice, and at an advanced age, oligodendrocyte transcriptomic changes are prominent in *Prnp* null mice.

### Neuronal AD-related transcriptome changes are PrP^C^-dependent while microglia are not altered

**Fig. 10:**
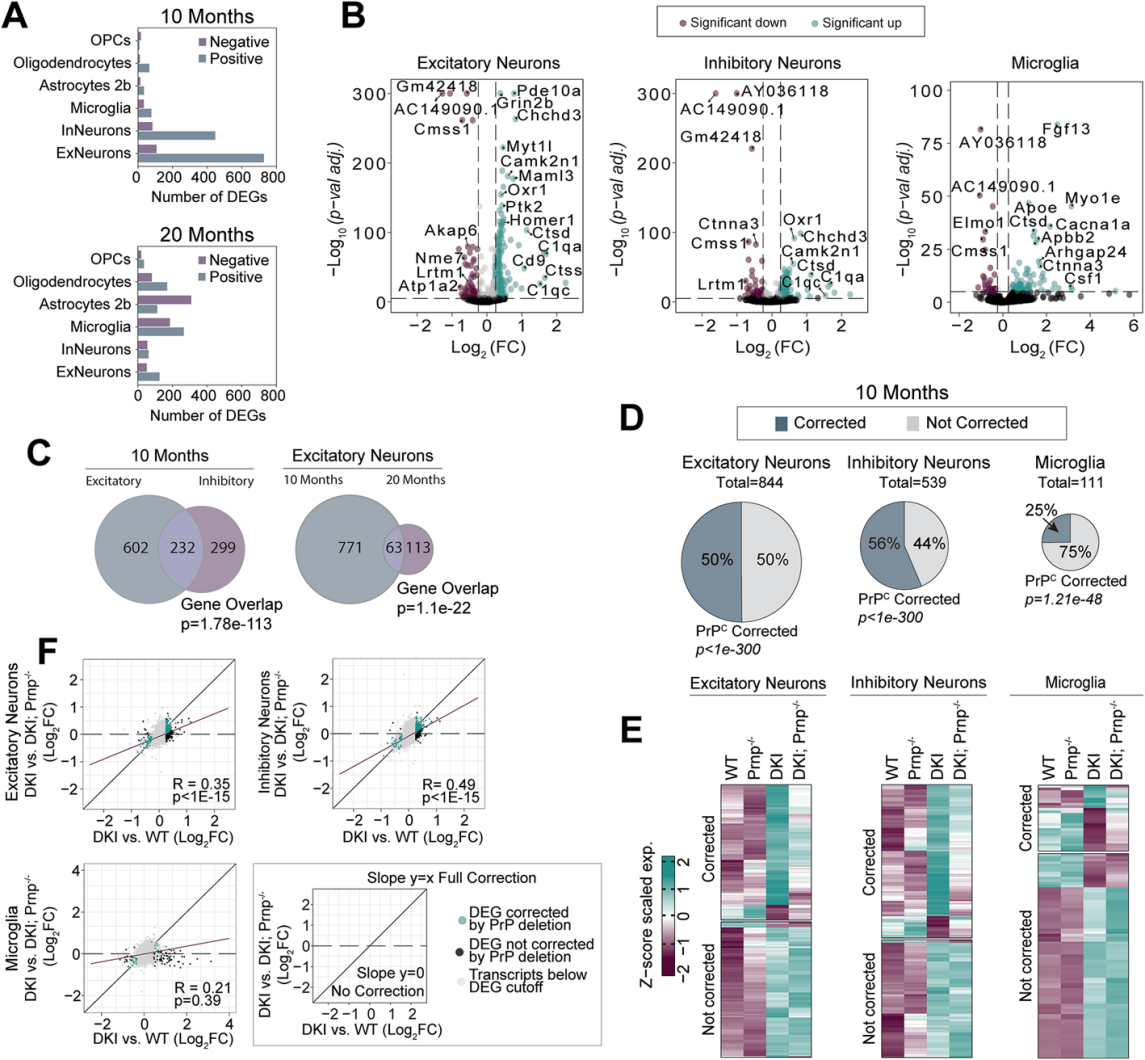
*Prnp*-dependence of cell type specific gene expression in DKI mouse model. **A** The number of AD-associated DEGS in neuronal and glial cell population at 10 (top) and 20-months (bottom) old DKI mice. **B** Volcano plots of excitatory and inhibitory neurons, and microglia at 10 months. Colored points indicated significant up or downregulated genes in DKI samples as compared to WT. **C** Venn diagrams showing the number of shared DEGs between excitatory and inhibitory neurons at 10 months, excitatory neurons populations at 10 and 20 months. The statistical significance of gene overlap was assessed by Fisher’s Exact test. **D** Pie charts illustrating the percentage of DEGs that are fully corrected by PrP^C^ gene deletion (Fisher’s exact test); the size of the chart is relative to total number of DEGs. **E** Heatmaps showing single DEG expression within each cell type, separated by corrected and non-corrected genes as represented in (**D**, **F**). DEG lists showing the remaining cell types are included in Supp. Table S2. **F** Transcriptome-wide correction by PrP^C^ gene deletion in neuronal and glial cell types. Cell type-specific comparison of AD-associated DEGs and PrP^C^-corrected DEGs in DKI samples. Log_2_FC between DKI and WT samples (AD effect) is plotted along the *x-*axis. Log_2_FC between DKI and DKI; *Prnp*^*−/−*^ samples (PrP^C^ effect) is plotted along the *y-axis*. Black points represent genes with Log_2_FC > 0.25 and *p* < *0.005*. Colored points represent DEGs that were corrected by PrP^C^ deletion. Points along the identity line (*x* = *y*) represent genes with equivalent differential expression between DKI; *Prnp*^*−/−*^ and WT, relative to DKI, indicating complete rescue by PrP^C^ deletion. Points along the line “*y* = 0” reflect genes unaffected by PrP^C^. *P*-values represents the significance of a non-zero linear regression relationship. The regression line (Pearson’s correlations, purple) represents transcriptome-wide effects of PrP^C^

Having assessed the baseline effect of PrP^C^ loss, we turned to the DKI model and searched for AD-related transcriptomic changes dependent on *Prnp* expression (Fig. 10). Our previous work had identified numerous neuron-specific DEGs rescued to a normal expression pattern by treatment with BMS-984923, a silent allosteric modulator of mGluR5 [19]. Based on PrP^C^ interaction with mGluR5 [18, 21], we hypothesized a related pattern here. Consistent with our previous findings and other studies, we observed numerous AD-associated DEGs in DKI compared to WT. At 10 months during the early phenotypic stage, the number of DEGs are considerably higher in neurons as compared to glial cell types (Fig. 10A, Suppl Table S1). Conversely, AD-associated gene expression changes in glial cells were more numerous at 20 months during disease progression as compared to 10 months (Suppl. Fig. S9A, B). In neurons, the number of DEGs was greater in excitatory than inhibitory neurons, though many DEGs were shared between excitatory and inhibitory neurons (Fig. 10B, C). The list of neuronal DEGs included upregulated *Camk2n1*, *Grin2b*, and *Chchd3*, genes essential for proper synaptic maintenance and stability, and for which dysregulation has been associated with AD pathology. Of note, neuronal DEGS are more numerous at the 10-month phenotypic initiation timepoint than at 20 months (Fig. 10C). Thus, this DKI model is characterized by early changes across neuronal cell types and delayed changes in multiple glial cell types.

**Fig. 11:**
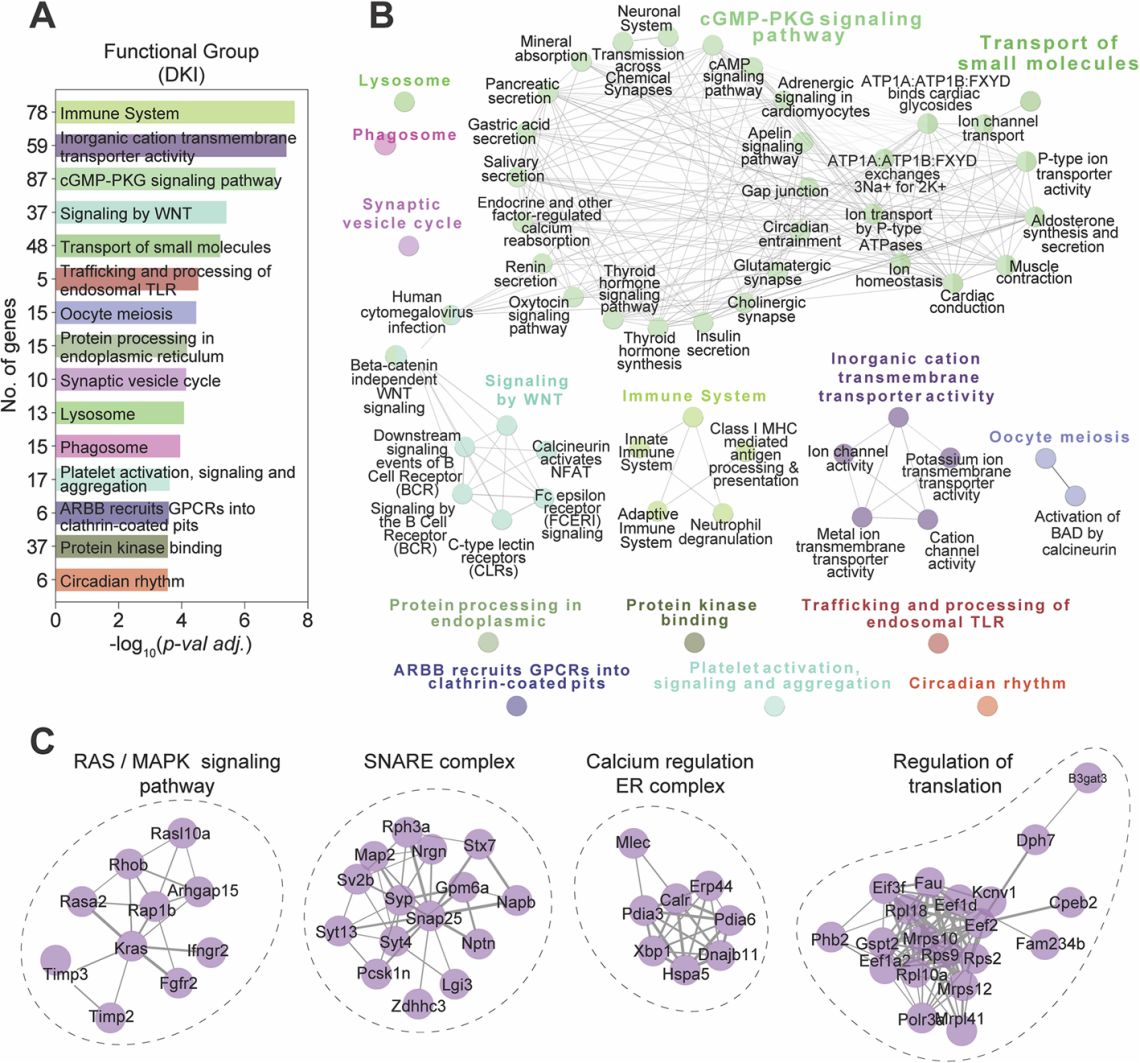
Pathways expression-normalized in DKI neurons by loss of PrP^C^ function. Functional enrichment analysis of AD-associated DEGs (DKI versus WT) corrected by *Prnp* deletion in pooled excitatory and inhibitory neuron samples. **A** Leading terms are color highlighted with corresponding *p*-values and total gene association count of each function group is graphed. **B** Gene set enrichment analysis against GO Molecular Function terms, KEGG and REAC Pathways, organized into functional network groups (nodes) based on shared gene associations (edges). **C** Functionally enriched PPI networks (encircled) with genes having stronger associations connected by thicker lines and genes with lesser or unknown associations not shown

Having established a temporal profile for transcriptomic response to the AD-related gene knock-ins, we examined whether *Prnp* deletion would correct the dysregulation in DKI; *Prnp*^−/−^ neurons, normalizing expression levels to WT levels. Indeed, for excitatory and inhibitory neurons there was a 50% and 56% correction of DEGs, respectively, in response to *Prnp*-deletion (Fig. 10D-E). In contrast, only 25% of genes were corrected by *Prnp* deletion in microglia at 10 months. To understand the transcriptomic-wide effect of *Prnp* deletion in our DKI model of AD, we plotted the gene Log_2_ fold-change (Log_2_FC) of DKI vs WT (AD-effect, *x-axis*) against that of DKI vs DKi; *Prnp*^−/−^ (deletion effect, *y-axis*). Using linear regression analysis on all plotted genes, a linear regression with slope = 1 represented full correction, and with slope = 0 corresponded to no correction of the overall AD-associated transcriptomic profile (Fig. 10F). Both excitatory and inhibitory neurons had a higher slope of regression (purple line) with corrected genes (teal) falling on the regression line of full correction (black). The linear regression was significantly different from a slope = 0 (*p* < *1E-15*) in both neuron types, again indicating a similar functional effect of *Prnp* across neuronal cell populations. In contrast, for microglia, the regression line slope was shallow and not significantly different from 0 (*p* = 0.39). Thus, *Prnp* has little transcriptomic effect on microglia cells at 10 months (Fig. 10F). To characterize the role of *Prnp* in DKI neurons, we performed functional and pathway analyses using the pooled gene set list of *Prnp*-corrected neuronal DEGs. Top functionally enriched networks include the cGMP-PKG, WNT, and RAS/MAPK pathways (Fig. 11A-C, Suppl Table S2). These are all second-messenger signaling pathways involved in the modulation of synaptic transmission and long-term potentiation. Thus, *Prnp* is required for early AD-related disruption of these neuronal pathways.

With regard to glial cells, the number of DEGs in DKI as compared to WT is higher at 20 months (Fig. 10A). We considered whether the temporal differences were related to a simple delayed onset or a shift in the expression pattern of glial reaction. We assessed whether gene expression changes at 20 months were also present at 10 months (Suppl. Fig. S9). In general, top DKI glial cell DEGs were uniquely present at 20 months, with little to no altered expression at 10 months (Fig. S9A). The notable exception was in microglia, for which most AD-associated DEGs had similar expression patterns at both 10 and 20 months (Suppl. Fig. S9B). In this regard, microglia are part of the disease initiation phenotype, while astrocytic and oligodendrocyte populations participate more specifically in later disease progression. Interestingly, lipoprotein genes *Apoe* and *Apod*, proteolytic protein genes *Ctsb*, *Ctsd*, and *Ctsl*, as well as complement gene *C1q* are also differentially expressed in both neurons and oligodendrocytes at 10 months.

Transcriptomic changes occur earlier and are stronger in microglia than in oligodendrocytes and astrocytes (Suppl. Fig. S9B, D). However, the functional effect of *Prnp* differed between glial cell types. At 20 months, we found that 26% of oligodendrocyte and 32% of astrocyte DEGs were corrected by *Prnp* deletion (Suppl. Fig. S9C). However, *Prnp* deletion only corrected 10% of AD-associated microglia DEGs. Transcriptomic-wide profile analysis of microglia had a linear regression slope of 0 (*p* = 0.39), indicating that *Prnp* has no direct role functional role in microglia (Suppl. Fig. S9D). Conversely, OPCs, oligodendrocytes, and astrocytes had transcriptomic profiles with linear regressions significantly different from a slope = 0. Although, there was the normalization of some AD-associated DEGs in oligodendrocytes and astrocytes cell populations of DKI; *Prnp*^−/−^ mice at 20 months, there was also genetic dysregulation with *Prnp* deletion alone when compared to WT (Fig. 8A). This is also clear in Venn diagrams of shared and unique DEGs between *Prnp*^*−/−*^, DKI, and DKI; *Prnp*^*−/−*^ as compared to WT (Suppl. Fig. S9E). Therefore, PrP^C^ appears to play a maintenance role in oligodendrocyte and astrocyte cell populations, as well as contributing to delayed and potentially indirect AD-related PrP^C^ signaling.

**Fig. 12:**
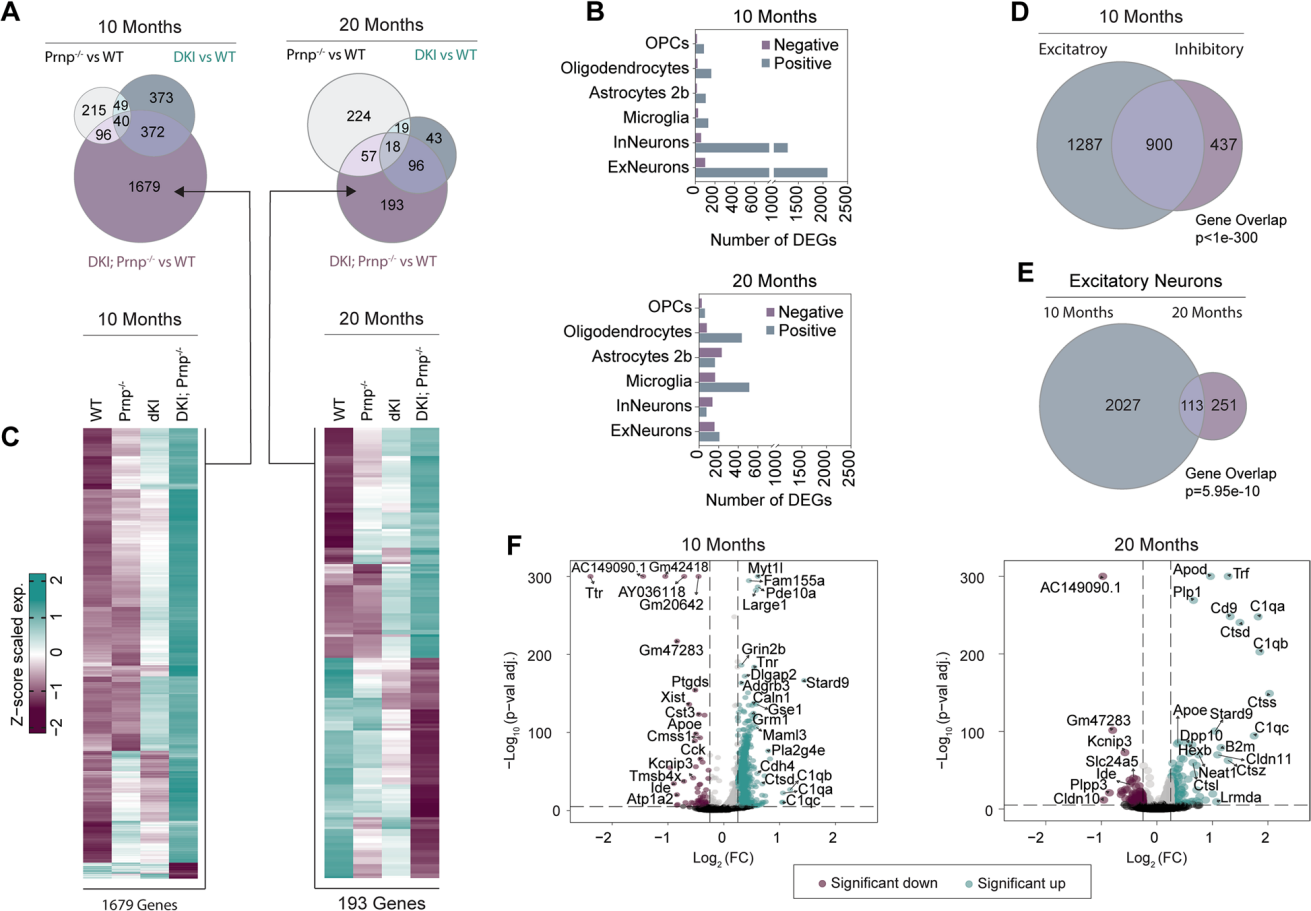
Synthetic gene expression profiles dependent on the interaction of DKI model with *Prnp* deletion. **A** Venn diagrams depicting the number of shared and unique DEGs between *Prnp*^*−/−*^, DKI, and DKI; *Prnp*^*−/−*^ samples in 10 (left) and 20 month (right) excitatory neuronal cell populations. **B** The number of positive and negative DEGs detected within neuronal and glial cell populations DKI; *Prnp*^*−/−*^ mice at 10 (left) and 20 months (right). **C** Heatmaps showing single DEG expressions of unique to DKI; *Prnp*^*−/−*^ samples (arrows) as represented in A. **D**, **E** Venn diagrams demonstrating the shared DEG significance (Fisher’s Exact Test) between excitatory and inhibitory neuronal cell population at 10 months (D) and between cell populations of excitatory neurons at 10 and 20 months. **F** Volcano plots showing DEGs of 10 (left) and 20 month (right) excitatory neuronal cell populations as measured by snRNA-seq comparing DKI; *Prnp*^*−/−*^ to WT samples

While investigating to what extent *Prnp* deletion could normalize AD-associated gene expression changes, we also found several dysregulated genes unique to the DKI; *Prnp*^*−/−*^ genotype (Fig. 12A). This DEG set is dependent on the synthetic interaction of *Prnp* loss with the AD model and is not detected with either single condition. The observed synthetic interaction was specific to neurons in the 10-month DKI; *Prnp*^*−/−*^ brain (Fig. 12B-F). The phenomenon was not observed in glial cells (Fig. 12B, Suppl. S9E). Specifically, the number of DEGs for each of the DKI; *Prnp*^−/−^ glial cell types were at levels similar to *Prnp*^*−/−*^ with many DEGs shared between the two genotypes (Suppl. Fig S9E). The 10-month neuronal synthetic interaction DEG set may reflect compensatory signaling mechanisms in DKI; *Prnp*^−/−^ neurons, so we performed gene ontology pathway enrichment analysis (Suppl. Fig S10, Suppl Table S2). Within the synthetic interaction set, nearly all enriched cellular compartment GO terms were associated with post-synaptic localization (Suppl. Fig. S8). With regard to functional pathway enrichment, the same glutamatergic and ionic conductance pathways associated with *Prnp*^−/−^ neurons were observed, but the top significant enrichment terms unique to the synthetic interaction set included Rho GTPase and cytoskeleton activity (Suppl. Fig. S10A-B). These Rho and cytoskeletal enrichment terms also had the most highly connected gene-network associations (Suppl. Fig. S10C). Collectively, our transcriptomic profiling results show that *Prnp* deletion normalizes DKI-dependent neuronal expression patterns specifically related to synaptic function and stability both by simple corrective rescue and by compensatory mechanisms.

## Discussion

The primary finding of the current study is that PrP^C^ mediates multiple neuronal AD-related phenotypes in mice with endogenous expression at the *Mapt* and *App* loci (*App*^*NL−G−F*^/*hMapt* double knock-in, or DKI). DKI mice demonstrate spatial memory deficits at 9 months that require a functional *Prnp* gene. Synapse loss, as measured by immunohistochemistry and SV2A PET, is also fully rescued by *Prnp* gene deletion and coincides with a significant decrease in synaptic tagging by C1q in DKI; Prnp^−/−^ mice. DKI mice demonstrate appreciable phospho-tau accumulation that is significantly reduced in the absence of PrP^C^. While *Prnp* deletion robustly rescued DKI behavioral and histological phenotypes at 9–10 months, we have not assessed the durability of these benefits at more advanced ages. The effects of *Prnp* gene deletion appear to be primarily neuronal, as evidenced by significant correction in DKI-dependent neuronal gene expression changes with *Prnp* gene deletion. In stark contrast, glial gene expression changes in DKI mice are only minimally altered by *Prnp* gene deletion, and microgliosis, astrocytosis, and Aβ levels are unchanged between DKI and DKI; *Prnp*^−/−^ mice. Several biomarkers of the essential role of PrP^C^ in neuronal AD phenotypes are translatable to clinical scenarios, including SV2A PET [27] and pThr217 levels [51], and these may be used to assess the therapeutic efficacy of targeting Aβo-PrP^C^ interaction in AD.

Neuronal metabotropic glutamate receptor 5 (mGluR5) is a transmembrane protein whose aberrant activation links the extracellular Aβo-PrP^C^ interaction to intraneuronal signaling in mouse models of AD [10, 18, 20, 21]. We recently demonstrated the efficacy of a silent allosteric modulator (SAM, BMS-984923) of mGluR5 signaling to rescue AD phenotypes in the DKI model [19]. Similar to *Prnp* gene deletion, SAM administration rescued synapse loss, reduced phospho-tau accumulation, and reduced synaptic tagging with C1q in DKI mice. Additionally, SAM corrected DKI-dependent genes primarily in neurons with little change observed in glial cells through transcriptomics and immunohistochemistry. The mirroring of SAM and *Prnp* deletion in rescuing DKI mice is consistent with their co-joined function as a molecular complex [18, 20, 21]. These data demonstrate that disrupting the Aβo-PrP^C^-mGluR5 pathway at two different steps rescues AD phenotypes, primarily through neuronal expression changes and with modulation of neuronal-glial interactions via C1q. The interaction of C1q with PrP^C^ may be direct or indirect, but is likely to occur at the surface of neuronal synapses. Despite the convincing similarity in mechanistic rescue of AD phenotypes with SAM administration and *Prnp* deletion, there are important differences in these two studies. The *Prnp* deletion study utilizes a constitutive knockout in a prophylactic mode prior to any AD related pathology, while the SAM treatment was effective in a therapeutic mode after the DKI mice were aged to the point of synapse loss (~ 12 month development here). Additionally, *Prnp*-null mice show transcriptomic changes separate from DKI effects, consistent with PrP^C^ having many purported physiological functions, including regulation of myelin maintenance, cell differentiation, and neuronal excitability [52]. In contrast, pharmacological targeting of mGluR5 with a SAM has minimal, if any, effect in WT mice [19, 20]. It remains possible that the perturbation of one of the many roles of PrP^C^ other than Aβo binding might contribute to the rescue of AD phenotypes in the DKI model. However, the well-described interaction of PrP^C^ with mGluR5 and mechanistic congruency with pharmacologically targeting mGluR5 suggest that the therapeutic effects of *Prnp* deletion on AD pathology are largely mediated through the Aβo-PrP^C^-mGluR5 pathway.

Further support for this hypothesis derives from the observation that deletion of *Prnp* normalizes DKI-dependent neuronal expression patterns, especially of genes whose products are localized to synapses. The neuronal DKI-dependent genes corrected by *Prnp* deletion have overlapping enrichment in pathways linked to several aspects of physiology, namely WNT/β-catenin signaling, immune signaling, endolysosomal trafficking, and cGMP- or cAMP-dependent Ca^2+^ signaling. The dysregulation of each of these pathways in neurons have been independently associated with AD pathogenesis. Specifically, impairments in ubiquitination pathways and endolysosomal trafficking have been implicated in AD-linked neurodegenerative paradigms contributing to synaptic dysfunction [53]. Dysregulated WNT/β-catenin signaling acts as a physiological switch between cell survival and cell death programs resulting from disruptions in DNA transcription and cellular ubiquitination processes. In the context of AD, upregulation of WNT signaling has also been linked to increased Tau phosphorylation, immune signaling, and eventual cell death and synapse loss [54, 55]. DKI-dependent dysregulated genes within the WNT/β-catenin signaling pathway include *Lgr4*, a known activator of WNT proteins and transcriptional regulators *Ppp3r1*, *Ppp25a*, and *Ppp3cb*. The expression of several V-ATPase genes (*Atp6v0b*, *Atp6v0e2*, *Atp6v1c1*, *Atp6v1g2*) involved in the lysosomal function, exhibit normalized gene expression levels in DKI; *Prnp*^−/−^ mice. Similarly, the dysregulation of V-ATPase components is mitigated by mGluR5 SAM treatment of AD mice [19]. The dysregulated expression of the endolysosomal genes, *Lamp1*, *Lamp2*, *Lgmn*, *Npc2*, *Ctsb*, and *Ctsl*, as well as *Fbxl12* and *Fbxl3*, are normalized in DKI; *Prnp*^−/−^ mice. *Skp1* and *Rbx1* are two genes involved in both WNT-directed ubiquitination and endosomal packaging, and they are dysregulated in DKI mice in a *Prnp*-dependent manner. The *Fbx* gene products are known interaction partners with *Skp1* and *Rbx1* proteins within ubiquitination ligase complexes but are also enriched in immune signaling pathways along with complement-related factors *B2m*, *Adara1*, *C4b*, and *Cd47*. In DKI mice, dysregulated genes involved in cellular calcium homeostasis, such as *Atp1a1*, *Atp1a3*, *Atp1b1* show normalized expression levels in the DKI; *Prnp*^−/−^ mice. Thus, multiple signaling pathways affecting calcium homeostasis, endolysosomal trafficking, and cell apoptotic pathways are features of AD pathology detected in DKI mice and require PrP^C^ expression.

Similar to SAM administration, in which only 7% of microglia DEGs were corrected with drug administration [19], *Prnp* deletion corrected only 25% of microglia DEGs in DKI mice. Meanwhile, *Prnp* deletion corrected greater than half of DEGs in excitatory and inhibitory neurons. As is consistent with previous studies investigating disruption of the Aβo-PrP^C^-mGluR5 pathway [14, 15, 19, 20], disease-associated changes in microgliosis, astrocytosis, microglial activation, total C1q levels, plaque load, and overall Aβ levels are unchanged. In the present DKI mouse study, activation of microglia appeared earlier and more robustly than activation of astrocytic cell types. Nonetheless, the transcriptomic signatures of glial activation matched closely to that of previously described disease-associated microglia (DAM) and astrocytic (DAA) upregulated gene patterns [56–58]. Genes such as *Apoe*, *Cd9*, *Cstd*, *Cstl*, *Aplp1*, which exhibit overlapping upregulation in DKI microglia and astrocytes have been reported in both human AD and mouse models of AD [59]. At 10 months, the activation profile of microglia coincides with the early genetic upregulation of *Apoe*, *Trem2*, *Tyrobp*, *Spp1*, and other DAM genes with simultaneous downregulation of genes such as *Tmem119*, *Cx3cr1*, and *P2ry12*. This transcriptomic pattern matches that of surveilling homeostatic microglia transitioning to a disease-associated state that is permissive to phagocytic engulfment of neuronal synapses, a process mediated by the *Trem2*-*APOE* signaling pathway [57]. In DKI mice there is progressive upregulation of DAA and DAM genes, with the respective astrocytic and microglia cell populations, and this occurs independently of PrP^C^. Despite glial gene expression and overall levels being largely unchanged, gial interaction with neurons appears to be modulated through PrP^C^-dependent neuronal transcriptomic changes and tagging of synapses by C1q. Thus, targeting PrP^C^ or mGluR5 pharmacologically rescues AD phenotypes through neuronal gene expression changes that abrogate the aberrant deleterious interactions between glia and neurons. Further investigations are necessary to understand these possible mechanisms, including specific C1q receptors and the molecular role of PrP^C^ in the localization of C1q to synapses with subsequent synaptic engulfment by microglia and/or astrocytes.

*Prnp*-null mice without DKI alleles exhibit phenotypes that underscore the broad and still partially understood physiological functions of PrP^C^. At 10 months age, mice with a targeted *Prnp* deletion demonstrated significantly reduced [^18^F]SynVesT-1 uptake in the olfactory bulb and increased uptake in the caudate-putamen. The importance of PrP^C^ in maintaining mature olfactory sensory neurons has been reported [38–40], and the loss of synapses as measured by SV2A PET further supports a function of PrP^C^ in proper olfactory tract development. The physiological role of *Prnp*-null specific synaptic density increases observed in caudate-putamen is not clear and merits future study. We did not conduct detailed assessment of motor function here. However, we observed no difference in swim speed or learning deficits between WT and *Prnp*^−/−^ mice. The rescue of DKI phenotypes by *Prnp* deletion appears specific to the context of pathologic Aβ and Tau.

Transcriptomic changes in *Prnp*-null mice were found primarily in excitatory and inhibitory neurons at 10 months age, with differentially expressed genes clustering in synaptic glutamate and ionic conductance pathways. PrP^C^ has been shown previously to have neuronal excitability-modifying properties [60], and Aβ has been shown to require PrP^C^ to inhibit ionic conductance [61]. PrP^C^ has also been shown to play a role in Ca^2+^ signaling through interactions with glutamate receptors [21, 62]. These studies are consistent with our observation of PrP^C^-dependent transcriptomic changes. However, distinct neuronal expression occurred in the DKI mice and their rescue by *Prnp* deletion was largely non-overlapping with PrP^C^-dependent changes on the WT background.

By 20 months age, expression of PrP^C^ altered oligodendrocyte transcriptomic patterns. PrP^C^ has been shown to play a role in oligodendrocyte differentiation and development, with oligodendrocyte precursor cells lacking PrP^C^ proliferating more vigorously and at the expense of differentiation [63]. Our results support a role for PrP^C^ in maintaining proper oligodendrocyte function, though this was not detectable until middle age. The difference between PrP^C^-dependent oligodendrocyte changes in 10-month-old mice compared to 20-month-old mice is dramatic. However, even at 20 months, there is no obvious behavioral change for WT mice lacking PrP^C^ that would suggest dysmyelination. While we also observed oligodendrocyte DEGs in DKI mice at 10 months and 20 months, the extent was far less than PrP^C^-dependent oligodendrocyte DEGs. Additionally, DKI-dependent DEGs occur more widely in cells like astrocytes and microglia at 20 months, while PrP^C^-dependent DEGs predominate in oligodendrocytes at 20 months.

Interestingly, a large group of neuronal DEGs were detected only as a synthetic phenotype of the knock-in/knock-out interaction of DKI; *Prnp*^−/−^ mice. Since behavioral and histological phenotypes are reduced in this condition, the synthetic interaction DEG subset may have a compensatory and beneficial effect to mitigate DKI-dependent changes and restore normal physiological function. At 10 months age, these genes were primarily observed in excitatory and inhibitory neurons, while at 20 months, microglia and oligodendrocytes showed the highest presence of synthetic interaction DEGs. Network analysis reveals that they are found in a variety of pathways not typically associated with disease pathogenesis. These include ribonucleotide binding, GTPase activator activity, and signaling by Rho GTPases. Further investigation into these genes, including a comparison with DKI mice with late conditional *Prnp* deletion or pharmacological inhibition of Aβo-PrP^C^ binding, should help elucidate their function in disease pathology and treatment. Notably, such synthetic interaction DEGs were not observed with mGluR5 SAM rescue of DKI mice [19].

Taken together, these data support the hypothesis that PrP^C^ may be a safe and viable target for pharmacological intervention in AD treatment. In fact, several potential therapeutics focused on disrupting the Aβo-PrP^C^-mGluR5 pathway have been shown to be preclinically efficacious. Anti-PrP^C^ antibody that crosses the blood–brain barrier and competitively inhibits Aβo oligomer binding has been shown to rescue impaired LTP, behavioral deficits, and synapse loss [14, 16, 17]. Similarly, a polymer that binds the Aβo-interaction site on PrP^C^ rescues several AD phenotypes and prevents hydrogel formation [15, 64]. mGluR5 has also been targeted through a silent allosteric modulator (SAM) which has shown strong preclinical promise and is currently in phase I clinical trials [19]. Methods described in this paper, including SV2A PET and phospho-tau profiling, can be used to evaluate the effects of these drugs in human subjects and understand any differences between human AD and mouse models.

Although endogenous expression at the *Mapt* and *App* loci potentially serves as a more accurate reflection of human AD progression than overexpressing Aβ models, there are limitations to our study design. The utilization of WT mice (*App*^+*/*+^, *Mapt*^+*/*+^*, Prnp*^+*/*+^*)* as a control group does not eliminate the possibility that *hMapt* contributes to observed phospho-tau accumulation independently of Aβ. It has been demonstrated that *hMapt* knock-in mice propagate pathological Tau species quicker than WT mice after injection with AD-derived tau [25]. However, in this same study, DKI mice also showed significantly increased phospho-tau accumulation compared to *hMapt* knock-in mice, suggesting at minimum an exacerbating role of Aβ in the formation of tau aggregates. Importantly, humanization of the *Mapt* gene does affect memory, neuroinflammation, and Aβ 40 and Aβ 42 levels on a wild type or *App*^*NL−G−F*^ background, supporting the hypothesis that Aβ production acts upstream to initiate Tau hyperphosphorylation. Our study aimed to specifically investigate PrP^C^-dependence in this double knock-in model rather than the changes individually associated with *hMapt* and *App*^NL−G−F^ knock-in. Here, the reduction in pathological tau aggregates with *Prnp* gene deletion, a high-affinity binding partner of oligomeric Aβ, supports our hypothesis that *Prnp* facilitates downstream neurotoxicity, including phospho-tau accumulation. Nonetheless, future studies comparing *App*^+*/*+^, *Mapt*^*hMapt/hMapt*^*, Prnp*^+*/*+^ mice to *App*^*NL−G−F/NL−G−F*^, *Mapt*^*hMapt/hMapt*^*, Prnp*^+*/*+^ mice may help to isolate the initiating effects of Aβ in this model as well as understand the extent to which other mechanisms or Aβ binding partners contribute to tau pathology.

## Conclusions

Our previous work has demonstrated the efficacy of targeting the Aβo-PrP^C^ interaction with anti-PrP^C^ antibody blockade, PrP antagonists, and gene knockout in mouse models overexpressing APP and Aβ. Here, we show that a functional *Prnp* gene is required for behavioral deficits and synapse loss in an AD mouse model with endogenous expression at the *App* and *Mapt* loci. Phospho-tau accumulation, a pathologic hallmark of AD that is limited or absent in other Aβ models, is detected in the DKI model and significantly reduced with targeted *Prnp* gene deletion. While single-nuclei transcriptomics reveal differential expression in neurons and glia of DKI mice relative to WT, glial DKI-dependent gene expression changes largely persist with *Prnp* deletion, consistent with unchanged histologic levels of microgliosis, astrogliosis, and Aβ levels between DKI and DKI; *Prnp*^*−/−*^ mice. DKI-dependent neuronal gene expression changes are significantly corrected with *Prnp* deletion, and corrected genes are primarily associated with synaptic function. These data support the efficacy of targeting the Aβo-PrP^C^ interaction to prevent Aβo-synaptotoxicity and pathologic tau accumulation in AD. The mechanisms by which PrP^C^ mediates AD-related phenotypes, including synaptic tagging by C1q, formation of dystrophic neurites, and phospho-tau accumulation, require further investigation. Importantly, the rescue of DKI mice by removal of PrP^C^ occurs despite continued Aβ plaque and glial rection, broadening therapeutic opportunities for AD.

### Supplementary Information

**Additional file 1: Supplemental Tables S1.** List of Differentially Expressed Genes. **Supplemental Tables S2.** Gene Set Enrichments for Differentially Expressed Gene lists. **Supplemental Figure S1.** Aβ(D54D2) accumulation in 3-month, 10-month, and 20-month-old DKI mice in hippocampus with time dependency. **Supplemental Figure S2.** Normal survival of DKI mice. **Supplemental Figure S3.** Limited locus coeruleus neuron loss in DKI mice. **Supplemental Figure S4.** Phospho-S396 tau accumulation in DKI mice localized to oligodendrocytes. **Supplemental Figure S5.** Prnp deletion does not alter Aβ accumulation in DKI mice. **Supplemental Figure S6.** Gliosis in DKI mice is unaffected by Prnp deletion. **Supplemental Figure S7.** Prnp deletion in DKI mice but reduces periplaque dystrophic neurites. **Supplemental Figure S8.** Cellular component analysis of DKI and Prnp-dependent DEGs in 10- month neuronal populations. **Supplemental Figure S9.** Glial cell activation in a DKI mouse model of AD. **Supplemental Figure S10.** Pathways with altered gene expression dependent on synthetic interaction of DKI model with Prnp deletion.

## Data Availability

Raw FASTQ sequencing files and gene count matrices have been deposited in NCBI’s Gene Expression Omnibus (GEO) under accession code GSE221712.

## Abbreviations

Astro: Astrocytes
cGMP: Cyclic guanosine monophosphate
DEGs: Differentially expressed genes
DKI: Double Knock-In, *App*^*NL*^^*−*^^*G*^^*−*^^*F/NL*^^*−*^^*G*^^*−*^^*F*^, *Mapt*^*hMAPT/hMAPT*^*, Prnp*^+^^*/*^^+^
DKI; * Prnp*^*−*^^*/*^^*−*^: *App*^*NL*^^*−*^^*G*^^*−*^^*F/NL*^^*−*^^*G*^^*−*^^*F*^, *Mapt*^*hMAPT/hMAPT*^*, Prnp*^*−*^^*/*^^*−*^
Epend: Ependymal
ExN: Excitatory neurons
FC: Fold change
GO: Gene Ontology
InN: Inhibitory neurons
MCL: Markov Cluster Algorithm
Micro: Microglia
Peri: Pericytes
PKGs: CGMP-dependent protein kinases
PPI: Protein-Protein Interactions
*Prnp*^*−*^^*/*^^*−*^: *App*^+^^*/*^^+^, *Mapt*^+^^*/*^^+^*, Prnp*^*−*^^*/*^^*−*^
Oligo: Oligodendrocytes
OPCs: Oligodendrocytes precursor cells
snRNA-seq: Single-nucleus RNA-sequencing
UMAP: Uniform manifold approximation projection
vLMCs: Vascular Leptomeningeal Cells
WT: Wild-type, *App*^+^^*/*^^+^, *Mapt*^+^^*/*^^+^*, Prnp*^+^^*/*^^+^
