# Supplementary material for: Neuronal transcriptome, tau and synapse loss in Alzheimer’s knock-in mice require prion protein
Source: Alzheimers Res Ther. 2023 Nov 15;15:201. doi: 10.1186/s13195-023-01345-z (PMC10647125; doi:10.1186/s13195-023-01345-z)
Supplement: Supplementary file 1 — Additional file 1: Supplemental Tables S1. List of Differentially Expressed Genes. Supplemental Tables S2. Gene Set Enrichments for Differentially Expressed Gene lists. Supplemental Figure S1. Aβ(D54D2) accumulation in 3-month, 10-month, and 20-month-old DKI mice in hippocampus with time dependency. Supplemental Figure S2. Normal survival of DKI mice. Supplemental Figure S3. Limited locus coeruleus neuron loss in DKI mice. Supplemental Figure S4. Phospho-S396 tau accumulation in DKI mice localized to oligodendrocytes. Supplemental Figure S5. Prnp deletion does not alter Aβ accumulation in DKI mice. Supplemental Figure S6. Gliosis in DKI mice is unaffected by Prnp deletion. Supplemental Figure S7. Prnp deletion in DKI mice but reduces periplaque dystrophic neurites. Supplemental Figure S8. Cellular component analysis of DKI and Prnp-dependent DEGs in 10- month neuronal populations. Supplemental Figure S9. Glial cell activation in a DKI mouse model of AD. Supplemental Figure S10. Pathways with altered gene expression dependent on synthetic interaction of DKI model with Prnp deletion. [file 13195_2023_1345_MOESM1_ESM.zip › Supplement.pdf]

Supplemental Material

Supplemental Tables S1-S2 Description and Supplemental Figures S1-S10.

Supplemental Tables S1. List of Differentially Expressed Genes.
Data from the experiment represented in main Figure 8 are presented. The data are separated by genotype, cell type and age. Genes meeting the defined threshold for differential expression relative to WT group of the same age, their respective Z-score, Log_2 Fold Change relative to WT, and the adjusted p-value are shown. Provided as Excel table.

Supplemental Tables S2. Gene Set Enrichments for Differentially Expressed Gene lists.
The neuronal DEGs listed in Suppl Table S1 were assessed for cellular compartment and functional pathway Gene Set enrichment using ClueGo. Three separate DEG lists were analyzed. The "*Prnp* Null" model utilized the DEG from *Prnp*$^{+/+}$ versus WT. The "*Prnp* corrected" model utilized those genes present the DEG list from DKI versus WT, but not in the DKI; *Prnp*$^{-/-}$ versus WT list. The "*Prnp* interaction DKI" model utilized DEGs found only in the DKI; *Prnp*$^{-/-}$ versus WT list. Separate tabs are provided for each model by Functional Pathway analysis and one tab for the cellular compartment analysis of all three models. Provided as Excel table.

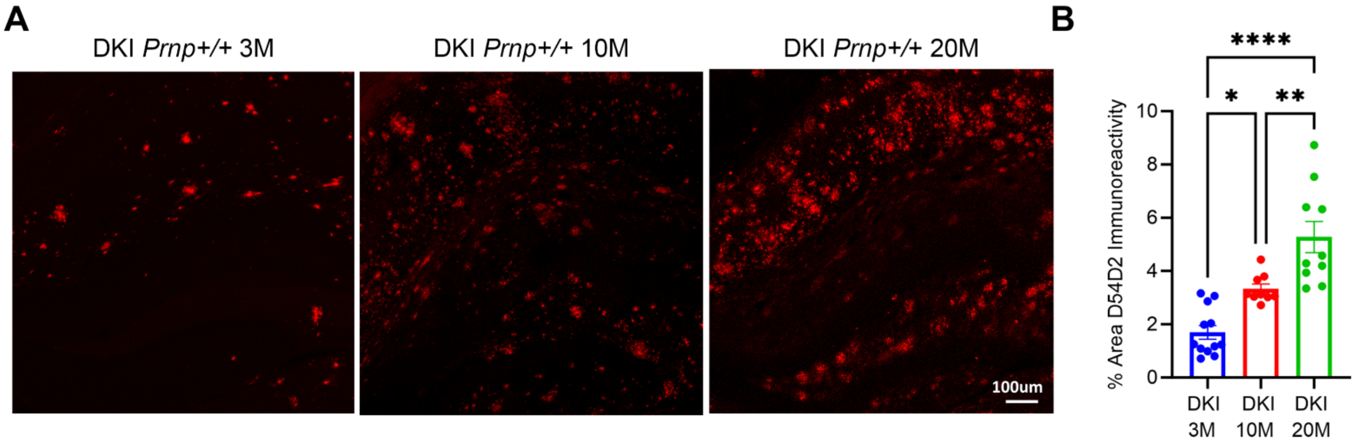

Supplemental Figure S1. Aβ(D54D2) accumulation in 3-month, 10-month, and 20-month-old DKI mice in hippocampus with time dependency.
(A) Representative images of Aβ (red) taken in the hippocampus of 3-month, 10-month, and 20-month-old DKI mice. Scale bar = 100 μm.
(B) Quantification of Aβ immunoreactive area in the hippocampus demonstrates a significant increase in Aβ deposition in older DKI mice compared to younger DKI, Data are graphed as mean ± SEM, analyzed by ordinary one-way ANOVA with Tukey's multiple comparisons test *P<0.05,**P<0.01, ****P<0.001, n= 12 for 3-month DKI, n= 8 for 10-month DKI , n= 10 for 20-month DKI.

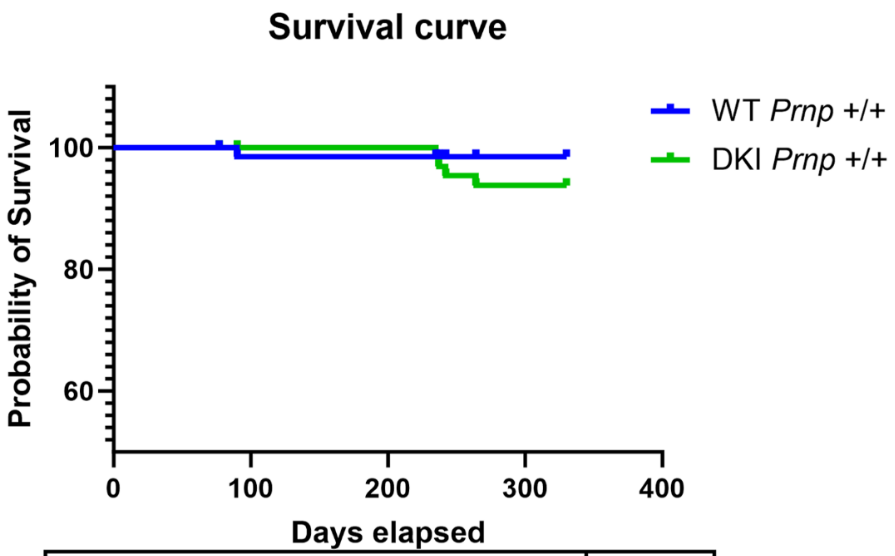

Log-rank (Mantel-Cox) test	
Chi square	1.800
df	1
P value	0.1797
P value summary	ns
Are the survival curves sig different?	No

Supplemental Figure S2. Normal survival of DKI mice. The survival rate remains at a similar level between WT *Prnp* +/+ and DKI *Prnp* +/+ mice. Data are graphed as survival, analyzed by Log-rank (Mantel-Cox) test, P>0.05 represent no significant differences between WT *Prnp*+/+ and DKI *Prnp*+/+ mice. n= 59 for WT *Prnp* +/+ group, n= 60 for DKI *Prnp* +/+ group.

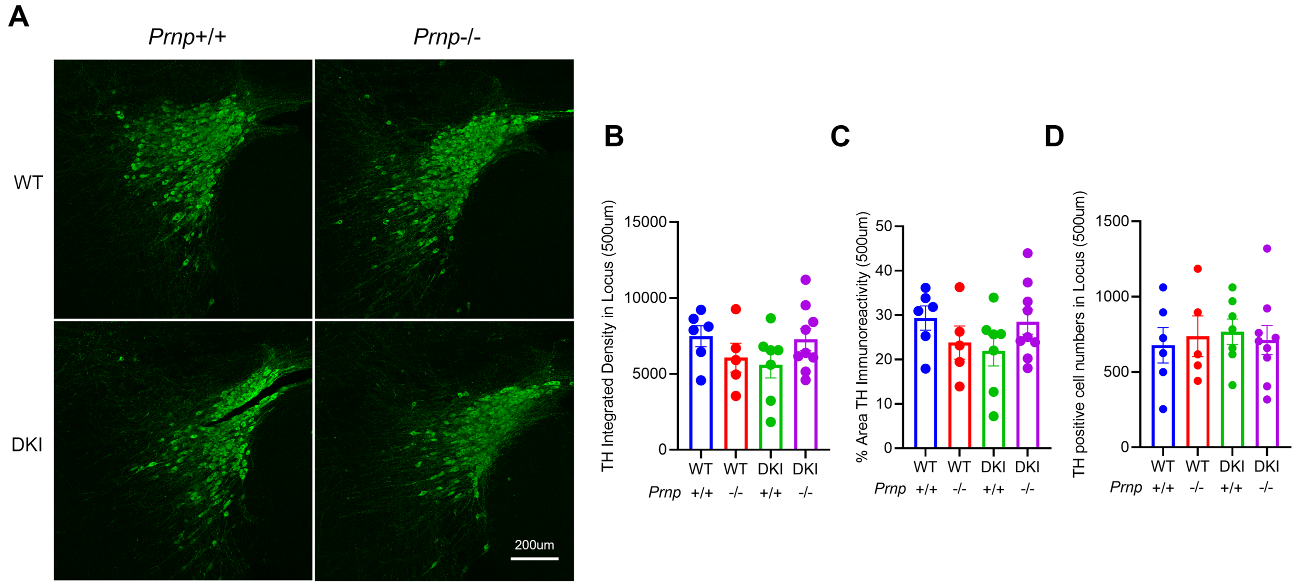

Supplemental Figure S3. Limited locus coeruleus neuron loss in DKI mice.

(A) Representative immunofluorescent images of anti-Tyrosine hydroxylase (TH) staining in locus coeruleus of 20-month-old WT, *Prnp*$^{-/-}$, DKI and DKI; *Prnp*$^{-/-}$ animals. Scale bar = 200 μm.

(B-D) Quantification of locus coeruleus TH immunoreactivity by evaluating TH integrated density (B), percentage of TH immunoreactive area (C), and TH positive cell numbers in locus coeruleus (D). There is a non-significant trend to decrease TH integrated density and TH immunoreactive area in DKI relative to WT. There is no numerical difference between WT and DKI; *Prnp*$^{-/-}$. Data are graphed as mean ± SEM, analyzed by ordinary one-way ANOVA with Dunnett's multiple comparisons test, P> 0.05, n=6 for WT, n=5 for *Prnp*$^{-/-}$, n=7 for DKI, and n=9 for DKI; *Prnp*$^{-/-}$.

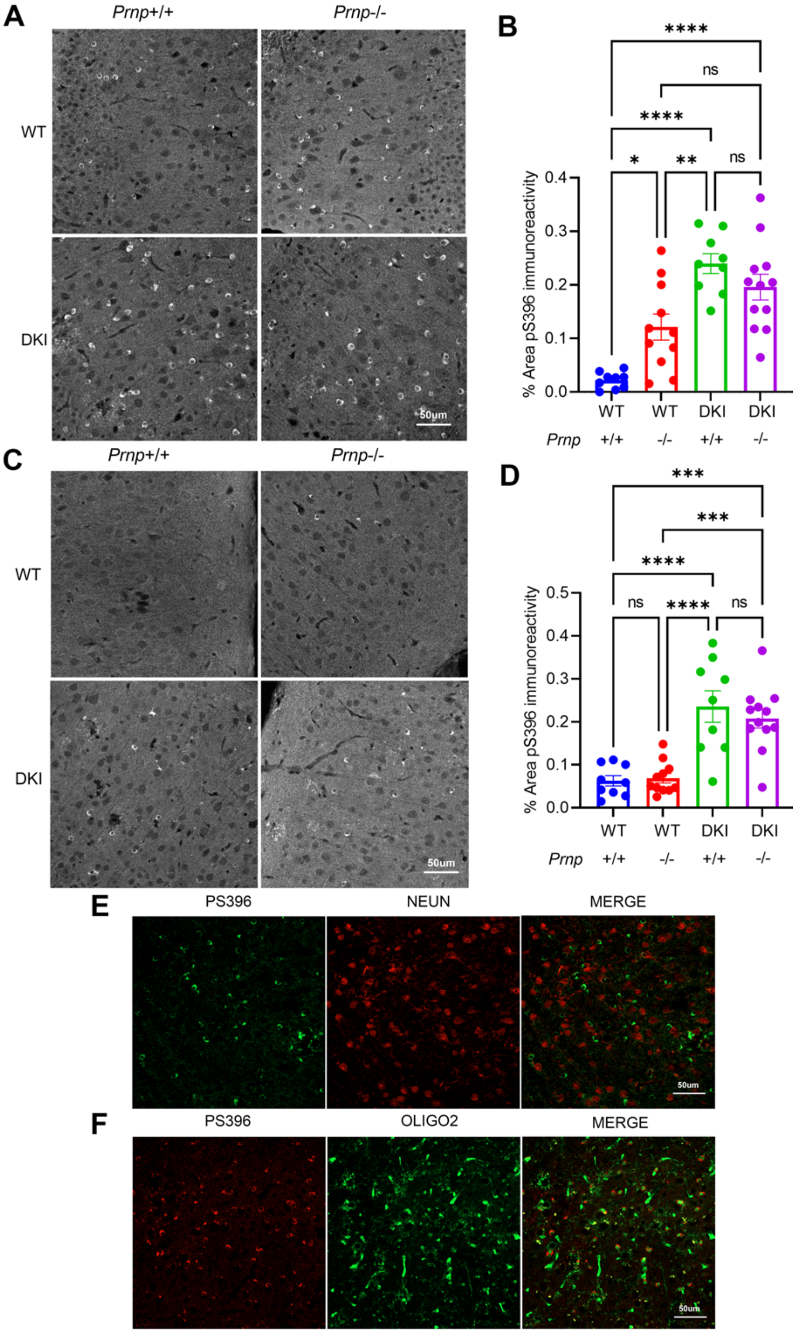

Supplemental Figure S4. Phospho-S396 tau accumulation in DKI mice localized to oligodendrocytes.

(A) Representative images of anti-phospho-S396 tau immunostaining from the retrosplenial cortex of 10-month-old WT, *Prnp*$^{-/-}$, DKI and DKI; *Prnp*$^{-/-}$ mice. Scale bar = 50 µm.

(B) Quantification of pS396 immunoreactive area from sections as in A demonstrates a significant increase for pS396-tau accumulation in *Prnp*$^{-/-}$, DKI and DKI; *Prnp*$^{-/-}$ brain relative to WT. The DKI and DKI; *Prnp*$^{-/-}$ levels are indistinguishable. Data are graphed as mean ± SEM, analyzed by ordinary one-way ANOVA with Tukey's multiple comparisons test, *P<0.05,**P<0.01, ****P<0.0001, n=9 for WT, n=11 for *Prnp*$^{-/-}$, n=9 for DKI, and n=12 for DKI; *Prnp*$^{-/-}$.

(C) Representative images of anti-phospho-S396 tau immunostaining from the auditory cortex of 10-month-old WT, *Prnp*$^{-/-}$, DKI and DKI; *Prnp*$^{-/-}$ mice. Scale bar = 50 µm.

(D) Quantification of pS396 immunoreactive area from sections as in C demonstrates a significant increase for pS396-tau accumulation in *Prnp*$^{-/-}$, DKI and DKI; *Prnp*$^{-/-}$ brain relative to WT. The DKI and DKI; *Prnp*$^{-/-}$ levels are indistinguishable. Data are graphed as mean ± SEM, analyzed by ordinary one-way ANOVA

with Tukey's multiple comparisons test, ***P<0.001, ****P<0.0001, n=9 for WT, n=11 for *Prnp*$^{-/-}$, n=9 for DKI, and n=12 for DKI; *Prnp*$^{-/-}$.
(E) Representative immunostained images of pS396 (green) and NeuN (red) taken in the medial cortex of 10-month-old DKI mice. pS396 distribution in DKI mice is not localized to neurons. Scale bar = 50 μm.
(F) Representative immunostained images of pS396 (red) and Olig2 (green) taken in the medial cortex of 10-month-old DKI mice. The pS396 signal in DKI mice is localized almost exclusively to oligodendrocytes. Scale bar = 50 μm.

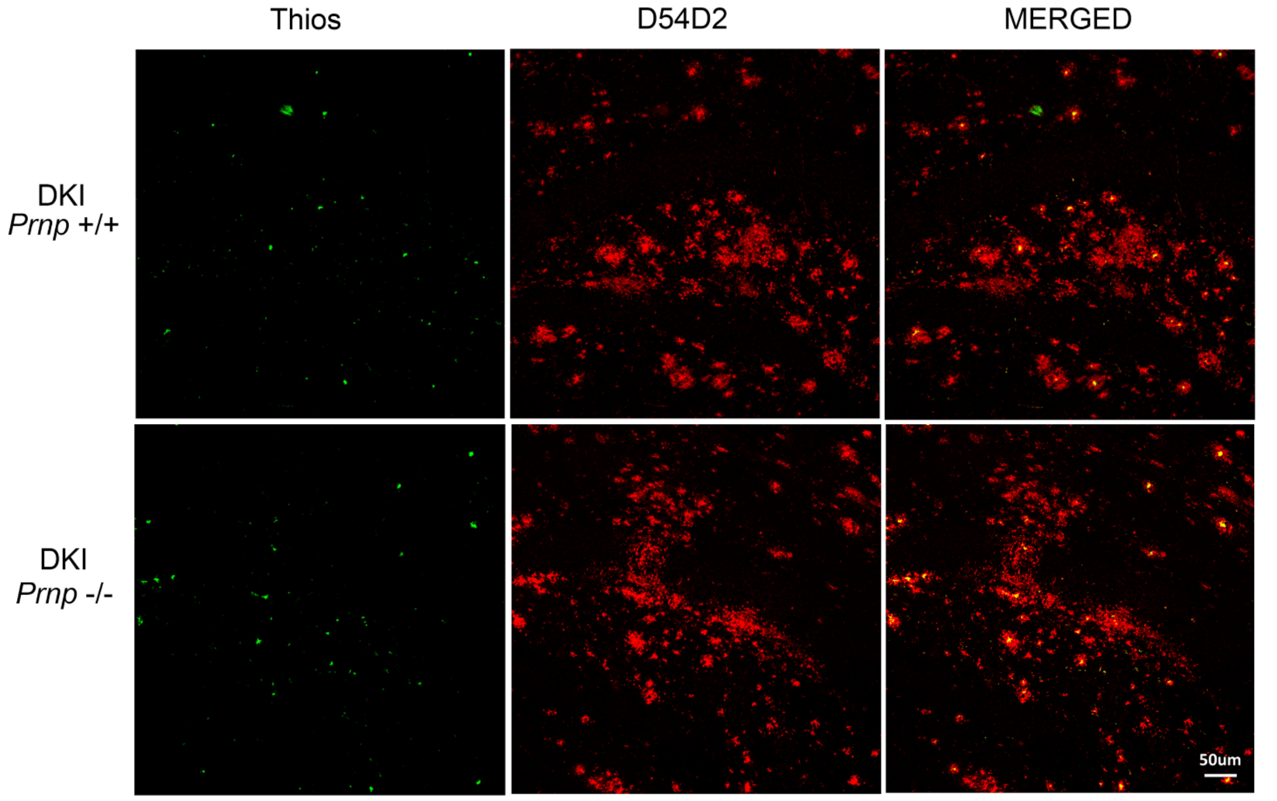

Supplemental Figure S5. *Prnp* deletion does not alter Aβ accumulation in DKI mice. Related to main Fig. 6A-C but shown with higher magnification and separate channels. Aβ accumulation is not altered by *Prnp*-/- deletion in DKI mice.

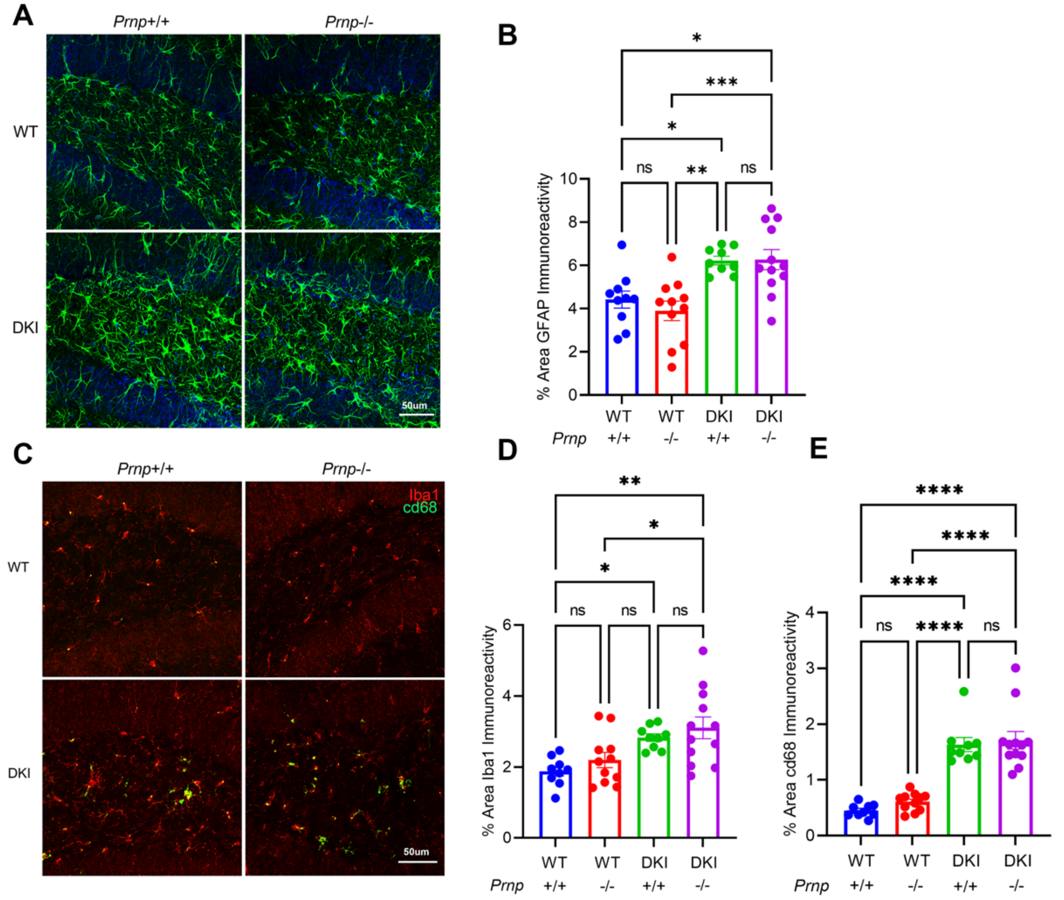

Supplemental Figure S6. Gliosis in DKI mice is unaffected by *Prnp* deletion.
(A) Representative images of GFAP (green) and DAPI (blue) taken in the hippocampus of 10-month-old WT, *Prnp*$^{-/-}$, DKI and DKI; *Prnp*$^{-/-}$ mice. Scale bar = 50 µm.
(B) Quantification of GFAP immunoreactive area in the hippocampus demonstrates a significant increase in astrocytosis for DKI mice compared to WT, but no effect *Prnp* of knockout. Data are graphed as mean ± SEM, analyzed by ordinary one-way ANOVA with Tukey's multiple comparisons test *P<0.05,**P<0.01, ***P<0.001, n= 10 for WT, n= 11 for *Prnp*$^{-/-}$, n= 9 for DKI, and n= 12 for DKI; *Prnp*$^{-/-}$.
(C) Representative images of Iba1 (red) and CD68 (green) taken in the hippocampus of 10-month-old WT, *Prnp*$^{-/-}$, DKI and DKI; *Prnp*$^{-/-}$ mice. Scale bar = 50 µm.
(D) Quantification of Iba1 immunoreactive area in the hippocampus demonstrates a significant increase in microgliosis for DKI mice compared to WT, but no effect *Prnp* of knockout. Data are graphed as mean ± SEM, analyzed by ordinary one-way ANOVA with Tukey's multiple comparisons test *P<0.05,**P<0.01, n= 9 for WT, n= 11 for *Prnp*$^{-/-}$, n= 9 for DKI, and n= 12 for DKI; *Prnp*$^{-/-}$.
(E) Quantification of cd68 immunoreactive area in the hippocampus demonstrates a significant increase in activated microglia for DKI mice compared to WT, but no effect *Prnp* of knockout. Data are graphed as mean ± SEM, analyzed by ordinary one-way ANOVA with Tukey's multiple comparisons test ****P<0.0001, n= 9 for WT, n= 11 for *Prnp*$^{-/-}$, n= 9 for DKI, and n= 12 for DKI; *Prnp*$^{-/-}$.

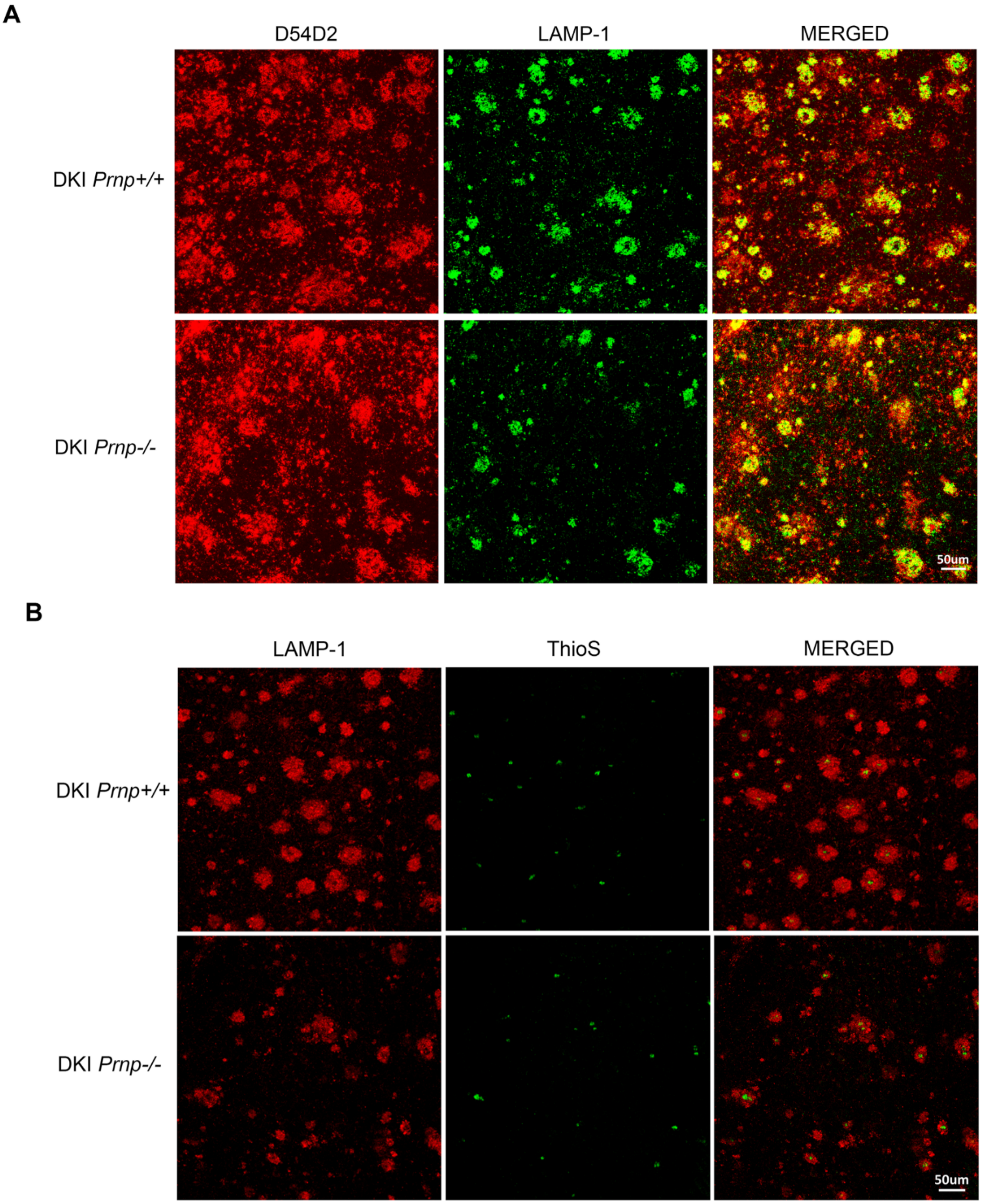

Supplemental Figure S7. *Prnp* deletion in DKI mice but reduces periplaque dystrophic neurites. Related to main Figure 6D, 6E but shown with higher magnification and separate channels. Aβ plaque detected with anti-Aβ immunostaining in A and by thioflavin in B is not altered by *Prnp* deletion, but plaque-associated anti-LAMP1 staining of dystrophic neurites is reduced in the absence of PrP^C.

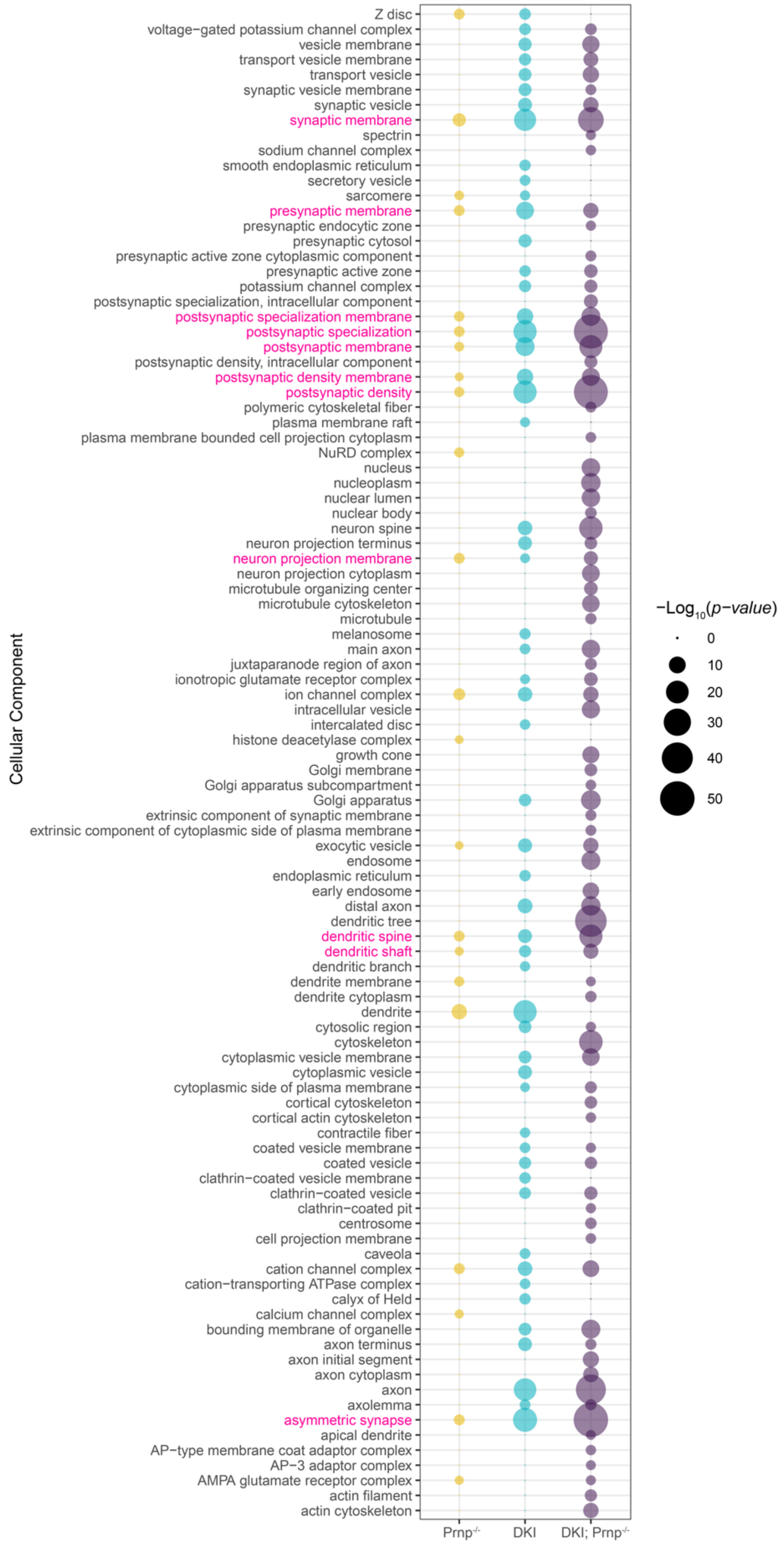

Supplemental Figure S8. Cellular component analysis of DKI and *Prnp*-dependent DEGs in 10-month neuronal populations. Dot plot of GO Cellular Component terms resulting from the enrichment analysis for DEGs of *Prnp*$^{-/-}$, DKI, and DKI; *Prnp*$^{-/-}$ samples as compared to WT. The size of dots indicates the P-value of significance for each term.

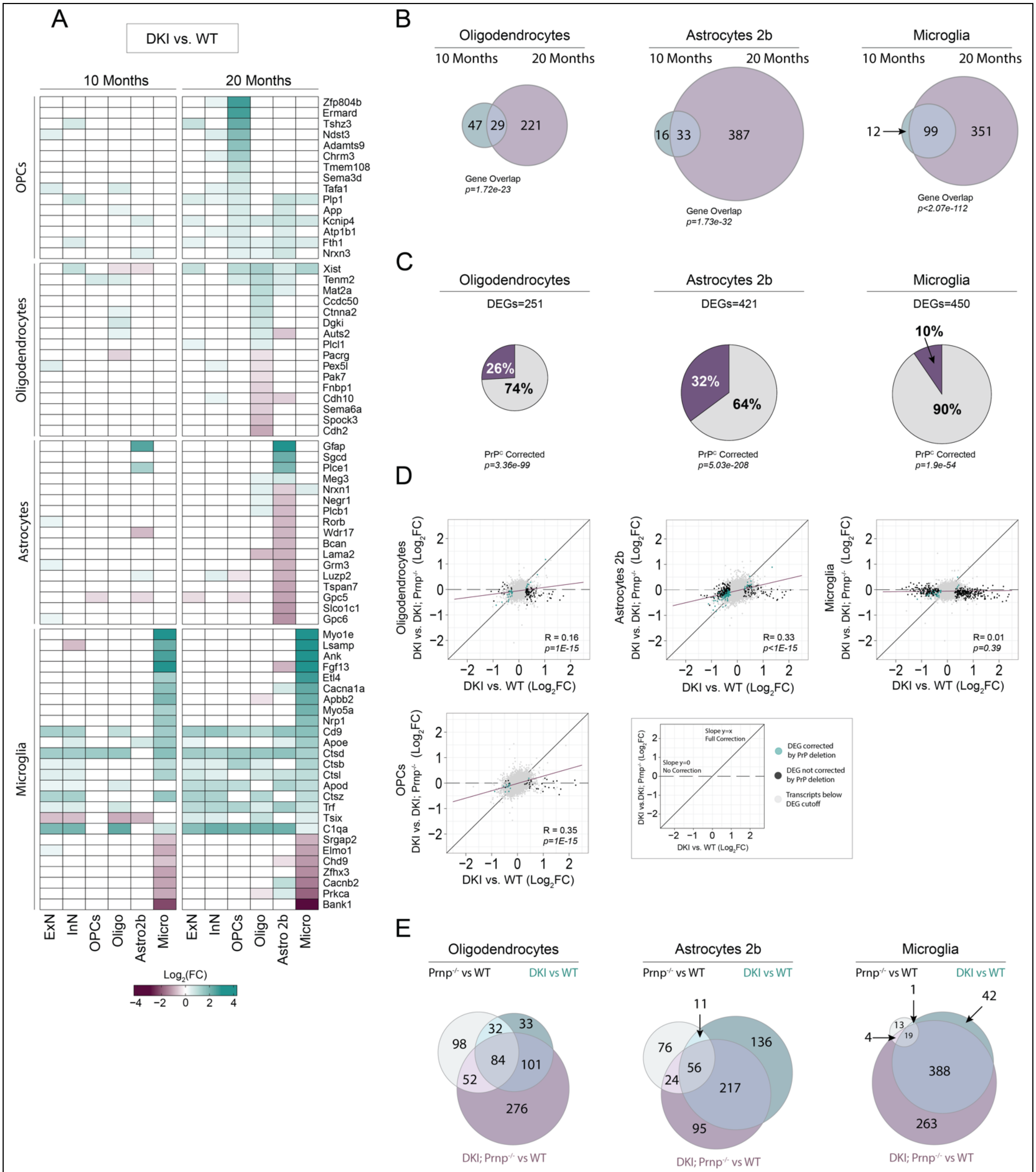

Supplemental Figure S9. Glial cell activation in a DKI mouse model of AD.

A-E, AD-associated transcriptional profile of OPCs, oligodendrocytes, astrocytes, and microglia cell populations comparing DKI versus WT samples.

A, Heatmap of the cellular expression of top differentially expressed glial gene markers at 10 and 20 months.

B, Venn Diagram comparing shared AD-associated DEGs shared by age-group and cell type.

C, Pie charts illustrating the percentage of AD-associated DEGs in glial cell populations corrected by PrP^C deletion.
B-C, Sizes of charts corresponds to the number of significant DEGs and statistical significance of gene overlap (Fisher's Exact Test) is the relative to number of DEGs.
D, Glial cell-type specific, transcriptome-wide comparisons of AD-associated DEGs and PrP^C-corrected DEGs. Log_2FC between DKI and WT (AD-effect) is plotted along the *x-axis*. Log_2FC between DKI and DKI; $Prnp^{-/-}$ (PrP^C-effect) is plotted along the *y-axis.* Black points represent significant DEGs with an absolute $Log_2(FC) > 0.25$ and *p-value <0.005*. Colored points represent DEGs also corrected by PrP^C deletion. Points along the identity line (x=y) represent genes with equivalent differential expression between WT or DKI; Prnp-/- relative to DKI, indicating complete rescue by PrP^C deletion. Points along the line "y=0" reflect genes unaffected by PrP^C deletion. The regression line (Pearson's correlations, purple) represents transcriptome-wide effects of PrP^C and *p-values* represents the statistical significance of a non-zero linear regression relationship.
E, Venn diagrams depicting the number of shared and unique DEGs between *$Prnp^{-/-}$*, DKI, and DKI; *$Prnp^{-/-}$* samples in glial cell populations at 20 months.

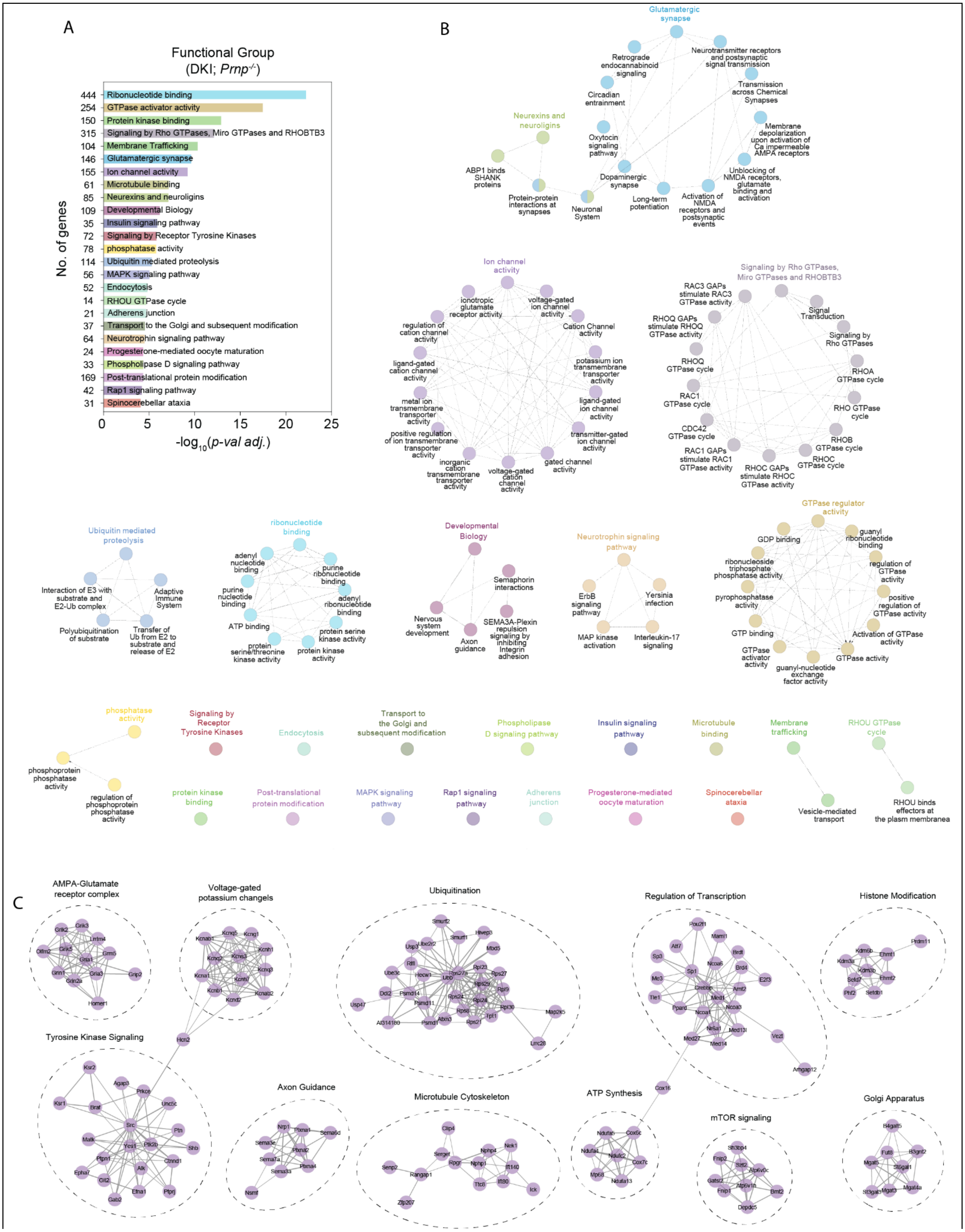
A
Functional Group
(DKI; Prnp-/-)
No. of genes
-log10(p-val adj.)
444 Ribonucleotide binding
254 GTPase activator activity
150 Protein kinase binding
315 Signaling by Rho GTPases, Miro GTPases and RHOBTB3
104 Membrane Trafficking
146 Glutamatergic synapse
155 Ion channel activity
61 Microtubule binding
85 Neurexins and neuroligins
109 Developmental Biology
35 Insulin signaling pathway
72 Signaling by Receptor Tyrosine Kinases
78 phosphatase activity
114 Ubiquitin mediated proteolysis
56 MAPK signaling pathway
52 Endocytosis
14 RHOU GTPase cycle
21 Adherens junction
37 Transport to the Golgi and subsequent modification
64 Neurotrophin signaling pathway
24 Progesterone-mediated oocyte maturation
33 Phospholipase D signaling pathway
169 Post-translational protein modification
42 Rap1 signaling pathway
31 Spinocerebellar ataxia
0 5 10 15 20 25
B
Glutamatergic synapse
Neurexins and neuroligins
Ion channel activity
Signaling by Rho GTPases, Miro GTPases and RHOBTB3
Ubiquitin mediated proteolysis
ribonucleotide binding
Developmental Biology
Neurotrophin signaling pathway
GTPase regulator activity
phosphatase activity
Signaling by Receptor Tyrosine Kinases
Endocytosis
Transport to the Golgi and subsequent modification
Phospholipase D signaling pathway
Insulin signaling pathway
Microtubule binding
Membrane trafficking
RHOU GTPase cycle
protein kinase binding
Post-translational protein modification
MAPK signaling pathway
Rap1 signaling pathway
Adherens junction
Progesterone-mediated oocyte maturation
Spinocerebellar ataxia
C
AMPA-Glutamate receptor complex
Voltage-gated potassium changels
Ubiquitination
Regulation of Transcription
Histone Modification
Tyrosine Kinase Signaling
Axon Guidance
Microtubule Cytoskeleton
ATP Synthesis
mTOR signaling
Golgi Apparatus

Supplemental Figure S10. Pathways with altered gene expression dependent on synthetic interaction of DKI model with *Prnp* deletion.

A-C, Functional pathway enrichment analysis of combined excitatory and inhibitory neuronal DEGs comparing DKI; *Prnp*$^{-/-}$ versus WT samples.

B, Pathway enrichment analysis (GO Molecular Functions, KEGG, and REAC Pathways) with terms (nodes) color coded and organized into functional grouping by gene associations (edges). The most significant leading term is color highlight with its corresponding P-value graphed in **A** along with the total unique gene association count with the respective functional network group.

C, PPI networks of gene associations grouped by highly enriched functional pathways. The number and thickness of lines represents stronger associations.
